# Olive Oil Industry By-Products as a Novel Source of Biophenols with a Promising Role in Alzheimer Disease Prevention

**DOI:** 10.3390/molecules29204841

**Published:** 2024-10-12

**Authors:** Marta Gonçalves, Marlene Costa, Fátima Paiva-Martins, Paula Silva

**Affiliations:** 1 School of Medicine and Biomedical Sciences (ICBAS), University of Porto, Rua de Jorge Viterbo Ferreira, 228, 4050-313 Porto, Portugal; mcgoncalves@icbas.up.pt; 2 REQUIMTE/LAQV, Department of Chemistry and Biochemistry, Faculty of Sciences, University of Porto, Rua do Campo Alegre, 687, 4169-007 Porto, Portugal; marlene.costa@fc.up.pt (M.C.); mpmartin@fc.up.pt (F.P.-M.); 3 Laboratory of Histology and Embryology, Department of Microscopy, School of Medicine and Biomedical Sciences (ICBAS), University of Porto, Rua Jorge Viterbo Ferreira, 228, 4050-313 Porto, Portugal; 4 iNOVA Media Lab, ICNOVA-NOVA Institute of Communication, NOVA School of Social Sciences and Humanities, Universidade NOVA de Lisboa, 1069-061 Lisbon, Portugal

**Keywords:** olive oil by-products, phenolic compounds, Alzheimer’s disease prevention, neuroprotection, Mediterranean diet, polyphenols, oleuropein, hydroxytyrosol

## Abstract

This review explores the potential health benefits and applications of phenolic secoiridoids derived from olive oil by-products in the prevention of Alzheimer’s disease (AD). As reviewed herein, polyphenols, such as epigallocatechin-3-gallate, epicatechin, and resveratrol, show in vitro and in vivo antioxidant, anti-inflammatory, and neuroprotective properties, and are particularly relevant in the context of AD, a leading cause of dementia globally. The olive oil industry, particularly in the Mediterranean region, produces significant amounts of waste, including leaves, pomace, and wastewater, which pose environmental challenges but also offer an untapped source of bioactive compounds. Despite promising in vitro and in vivo studies indicating that olive-derived polyphenols, such as oleuropein and hydroxytyrosol, may mitigate AD pathology, human clinical trials remain limited. The variability in extraction methods and the complex nature of AD further complicate research. Future studies should focus on standardizing the protocols and conducting robust clinical trials to fully assess the therapeutic potential of these compounds. This approach not only supports the development of new treatments for AD but also promotes environmental sustainability by valorizing olive oil industry waste.

## 1. Introduction

Olive oil is the main cooking oil used in the Mediterranean diet and stands out for its organoleptic and nutritional properties [1,2]. Its production is concentrated in the Mediterranean basin, although there are attempts to produce it on a large scale in other areas, such as Australia and the United States [1]. According to data estimated by the International Olive Council, around 2.4 million tons was produced worldwide in 2023/2024. The most important olive oil producer countries are concentrated in the Mediterranean area, namely Spain, which is the world’s largest producer with around 31.8% of production, followed by Italy (12%), Turkey (8.7%), Tunisia (8.3%), Greece (8.1%), Portugal (6.2%), Morocco (4.4%), and Syria (3.9%) [3]. Olive oil production is, therefore, of foremost importance for these countries. Spain, as the world leader in the production and export of olive oil [3], strongly influences the sector’s trade policies. A large variation in the production of olive oil has been observed in the last decade, with an important reduction in its production by Spain due to the drought of recent years [4,5].

Olive oil production generates high quantities of by-products in these countries, either from pruning or in the olive mills, including leaves, pomace residues, stones, and wastewater. These by-products bring major environmental problems, but they are also inexpensive raw materials containing bioactive molecules, which have potential uses as nutraceuticals or as additives to functional foods, packaging materials, and pharmaceutical formulations. Therefore, it is important to consider these potential uses for circular economy in the olive oil supply chain [6].

Since the Seven Countries Study developed in the 1950s, the association between the Mediterranean diet and a lower incidence of cardiovascular diseases has been established [7]. It has also become recognized that adherence to the Mediterranean diet has equally protective effects against depression and cognitive impairment [8]. Several of these beneficial effects have been attributed to the phenolic content of virgin olive oil (VOO) and it is now evident that the phenolic compounds contained in olive oil are fundamental to the effects of the Mediterranean diet on health. Moreover, olive by-products, such as leaves, fruits, seeds, and bark, have been used in traditional medicine since ancient times [9]. The major class of phenols present in olive trees is the secoiridoids, a class of phenols exclusively present in the Oleaceae family, which are iridoids derivatives formed by the cleavage of the iridoid cyclopentane ring and which contain a phenolic moiety arising from the phenylpropanoid pathway. These are secondary metabolites with an important role in the prevention of oxidative stress in the plant under the normal Mediterranean environment. The most important phenol of this class found in olive tree is oleuropein, and it can represent up to 14% (dry matter) of olives and olive leaf material [10]. The biological activity, such as the antioxidant, anti-hypertension, and anti-inflammatory properties, of several olive oil phenols have been reported [10,11,12]. However, although clinical studies consistently highlight the beneficial effects of the Mediterranean diet [7,8] the molecular mechanisms of action of extra virgin olive oil (EVOO) in the context of neurovascular diseases remain largely unexplored [13,14,15,16].

In this century, with the increase in life expectancy, neurologic disorders are becoming a growing public health concern [9], with Alzheimer disease (AD) as the leading cause of dementia, contributing to up to 80% of all dementia cases worldwide among individuals aged over 65 [17]. AD now represents more than 55 million cases globally, but it is projected to reach 152 million cases by 2050 [14]. Consequently, there is an urgent need for effective therapeutic management to prevent or arrest the disease’s progression. Considering that the multifactorial etiology of neurologic disorders appears to be a major concern, natural components can be considered as potential therapeutic options due to their multiple-target therapeutic properties, including antioxidant, anticancer, anti-diabetic and anti-inflammatory activities [9,11,12,14,18]. The generation of high amounts of inexpensive olive oil by-products by the olive oil industry may offer an opportunity to develop innovative and value-added products to be used in diets designed to prevent neurological disorders. This review provides an overview of the beneficial health effects, molecular mechanisms, and potential applications of phenolic secoiridoids from *Olea europaea* in AD prevention.

## 2. Olive Tree Bioactive Phenolic Compounds

**Figure 1:**
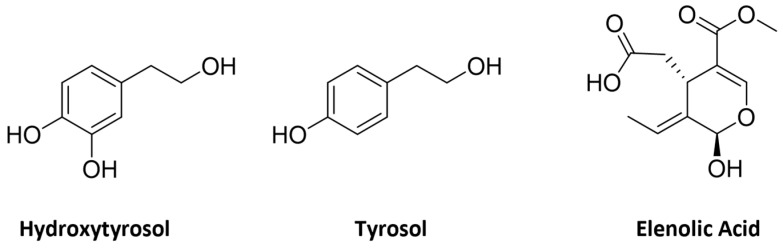
Structures of the main phenolic alcohols present in olive tree materials, as well as that of elenolic acid.

**Figure 2:**
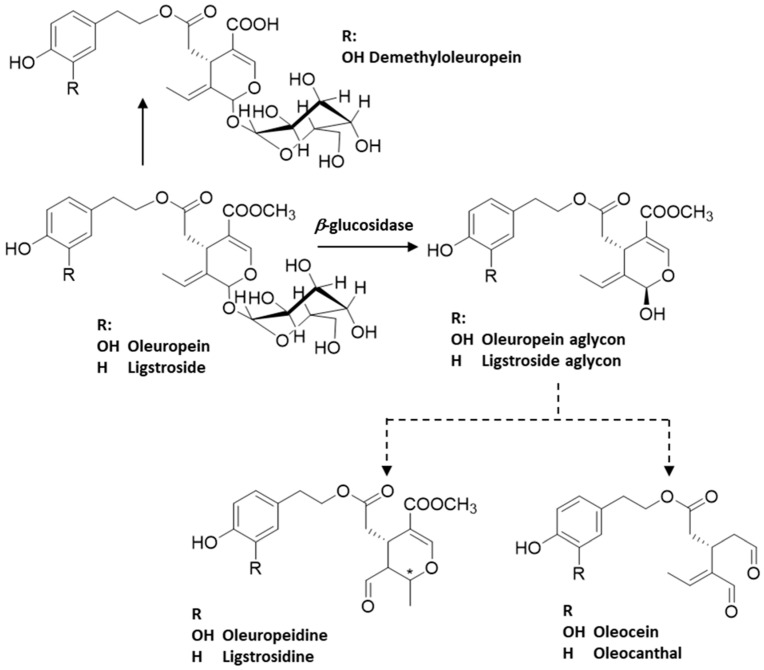
Structures of the most important olive oil phenolic secoiridoids [21].

Olive trees have a wide number of bioactive compounds. The fruit, which is the olive, exhibits a significant variety of secondary metabolites and is rich in tocopherols and carotenoids, which play crucial role in the protection of the oil against oxidative degradation [19]. It also possesses a valuable phenolic composition, comprising up to 3% of the fresh pulp weight, belonging to different classes, namely flavonoids, such as quercetin and luteolin, coumarins, lignans, like pynoresinol and acetopynoresinol, phenolic acids, phenolic alcohols, namely hydroxytyrosol (HT) and tyrosol (Figure 1), hydroxyl-isochromans, and secoiridoids, for instance the glycosides oleuropein and ligstroside (Figure 2) [20,21]. Olive secoiridoids are usually esters of elenolic acid or its derivatives (the secoiridoid moiety) and hydroxytyrosol or tyrosol (the phenolic moiety).

As previously mentioned, olives are particularly rich in secoiridoid phenolics [22], with the glycoside oleuropein being present at levels of up to 14% of their dry weight [10]. During the VOO mechanical extraction process, secoiridoid glycosides from olives, such as oleuropein and ligstroside, are hydrolyzed by *β*-glucosidases and methylesterases (Figure 2).

**Figure 3:**
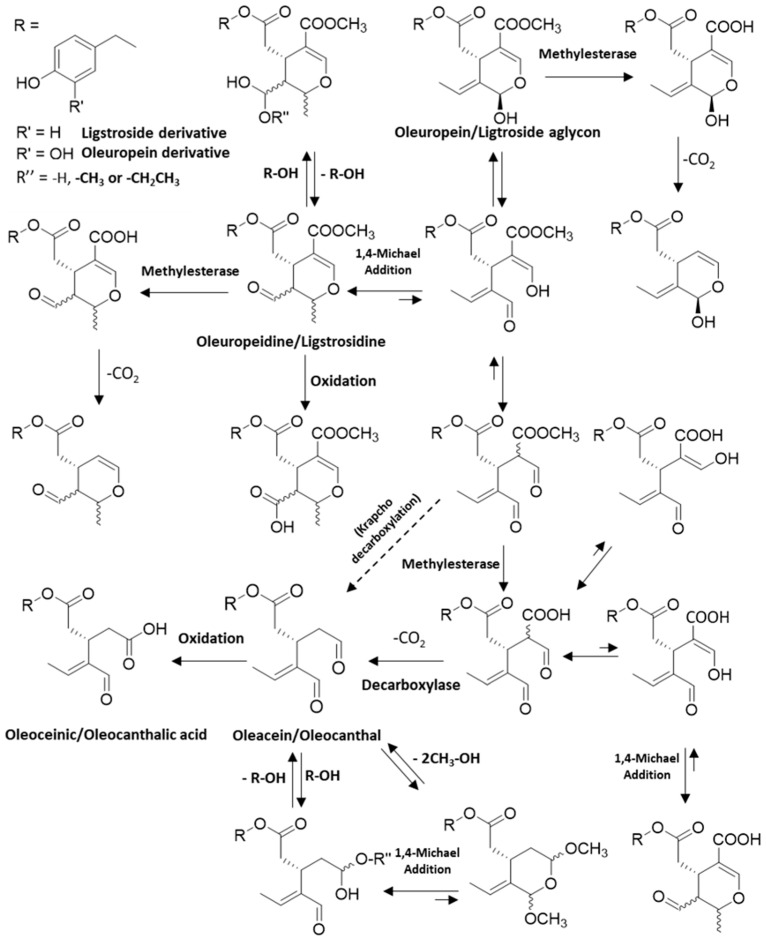
Structures of olive oil secoiridoid phenolic compounds and possible biochemical transformation pathways of oleuropein and ligstroside aglycones in olives, during olive oil extraction and during the extraction of olive oil phenolic compounds with ethanol/methanol [21].

These aglycones have a quite labile ring that easily opens and produces, by several chemical reactions, a complex mixture of more lipophilic aglycones, namely oleuropeidine, oleacein and oleocanthal (OC), that are released into the oil (Figure 2 and Figure 3) [21].

**Figure 4:**
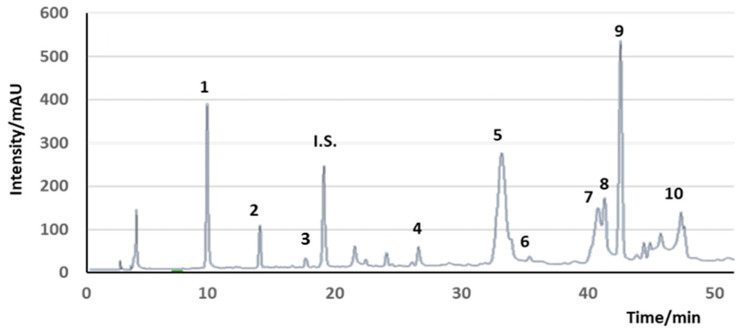
Example of a HPLC chromatogram of an extra virgin olive oil (EVOO) phenolic extract. 1, hydroxytyrosol; 2, tyrosol; 3, caffeic acid; 4, hidroxytyrosyl acetate; 5, oleacein; 6, oleuropein; 7, oleocanthal; 8, pynoresinol; 9, acetoxipynoresinol; 10, oleuropeidin; I.S., internal standard, syringic acid.

In contrast, during extraction, the more hydrophilic phenols, such as HT and oleuropein, tend to diffuse into the water phase [23]. Therefore, a considerable amount of phenolic compounds (98%) is lost in olive pomace (OP) and in the vegetarian water phase, and only around 2% of the more lipophilic phenolic compounds ends up in olive oil [23]. Consequently, the phenolic composition of olive oil and olives is often quite different (Figure 4) [24].

Although the phenolic compounds present in olives can also be found in any part of the olive tree, there are some differences in composition depending on which part of the plant we are referring to. Usually, the stems and branches exhibit a higher concentration of phenolic compounds, like taxifolin and oleuropein [25,26,27]. Moreover, some secoiridoid compounds are exclusively found in olive seeds, such as nüzhenide [28,29]. Nevertheless, it is necessary to keep in mind that the composition and concentration of secoiridoids and other phenolic compounds in olives, olive oil, and in any other olive tree tissue, are deeply dependent on the geographic origin, edaphoclimatic conditions, ripening degree, cultivar, and the cultivation and extractions methods applied.

## 3. Olive Oil Production and By-Products

### 3.1. Overview of Olive Oil Production

**Figure 5:**
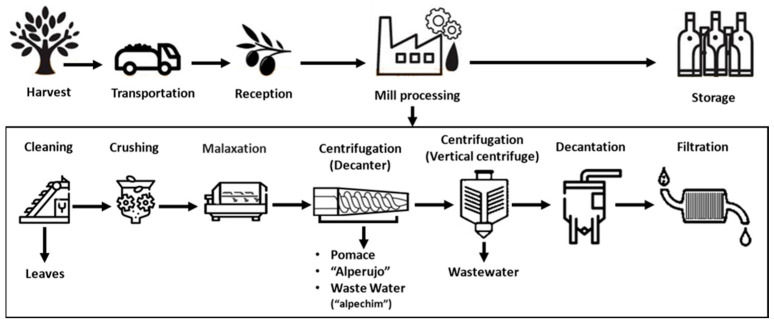
Overview of olive oil production.

EVOO, the natural juice of olives, has been known for centuries for its health benefits and as a key component of the Mediterranean diet, and is considered one of the healthiest edible fats. To produce high-quality EVOO, it is necessary to take care of the olive tree and olive grove throughout the year, both in terms of pest control and in terms of pruning, as the quality of the oil begins in the olive tree and in olive. One of the key factors to produce high-quality EVOO is the olive harvest, since olives need to arrive in good condition at the mill. Therefore, both the collection and transport to the oil mill must be performed on the very same day of harvest and with care so that the olive does not suffer any damage. Once they have arrived at the oil mill, the leaves are removed from olives by forced ventilation and the olives can undergo a quick wash, only if necessary, using clean water. Afterwards, and in the same day, olives are crushed to extract the oil [30]. The resulting paste from the crushing is subjected to a slow continuous kneading (malaxation process) in thermobilters, at temperatures below 27 °C, aimed at breaking the emulsions formed during the crushing process and facilitating oil droplet coalescence without the occurrence of oxidation and polyphenolic degradation. Next, the oil is usually separated from the olive paste by pressing using a discontinuous hydraulic pressing or by centrifuging the olive paste using a continuous horizontal centrifuge or decanter. In this operation, an oily must is separated from the solid part (olive pomace, OP) and from most of the vegetation water (“alpechin”) [30]. A new centrifugation, using a vertical centrifuge, is now applied to the oily must, separating the oil from all possible moisture and fine solids. After this centrifugation, the oil is separated from the water and the impurities (small olive particles) that have not been separated in the centrifugation processes by decantation. The oil then rests for a couple of hours in conical tanks so that the remaining microparticles and residual moisture are deposited, purged, and cleaned. Once the cleaned oil is obtained, it can be filtered so that the resulting oil is completely free of particles and stored in stainless steel tanks at 15–18 °C until packaging (Figure 5) [30].

Although most of the olive oil extraction process is similar in most olive oil industrial facilities, different methods can be applied in the separation of olive oil from the olive paste. Spain mainly uses the two-phase system decanter (~99%), but other countries, also with high production, still uses pressing systems (traditional) and three-phase system decanters. The three different extraction systems result in the production of different quantities of by-products. While the hydraulic press system generates between 33 and 40 kg of OP, containing ~25% of water, and 40–60 kg of wastewaters per 100 kg of processed olives, the use of a two-phase decanter produces around 80 kg of “alperujo”, a mixture of OP containing vegetation water (~60%), but only a small quantity of wastewater (~10 kg). The use of the three-phase decanters involves the addition of water to olive paste and, therefore, three phases are obtained: the oil phase (oily must), a solid phase (OP and stones, 40–57 kg/100 kg of processed olives, containing also a high amount of water, around 50%), and a vegetation water phase (~80–130 kg/100 g of processed olives). The drawbacks of this system are the production of substantial amount of wastewater or “alpechin”, and the leaching of polar compounds, such as polyphenols, with a negative impact on the environment [31] and lower enrichment of the oil in terms of the phenolic compounds.

### 3.2. Types of By-Products Generated by the Olive Oil Industry

#### 3.2.1. Olive Pomace

During harvest, over 12 million tons of OP are produced each year [32]. The appearance of this by-product is usually that of a pinkish mud, caused by the initial formation of quinones from polyphenols, that quickly oxidize to a brownish color [32,33]. It is composed of olive pulp and skin, crushed olive stones and seeds, oil, and water. This by-product is usually stored for long periods in large containers for evaporation [32,33] and both due to this long storage period and given the seasonality of this crop, it can act as a limiting factor in olive oil mills and may hinder the continuous production of high quality olive oil [32] given the difficulties in the management of such high amount of OP obtained in a short period of time.

After water evaporation, OP can still be used to extract OP oil [34,35] with the aid of organic solvents, such as hexane, and, afterwards, it can be refined and used in the production of edible non-VOO [34,35]. The resulting biowaste may now be used as biomass to produce fuel pellets or for animal feeding [36,37,38]. However, the need for transportation, pumps, and special storage tanks for the water evaporation of large amounts of OP demands high amounts of energy, and, therefore, increased financial resources [35,39]. Moreover, the possible formation of benzopyrenes during the drying process may limit the usage of OP oil as a food ingredient [32,35].

**Figure 6:**
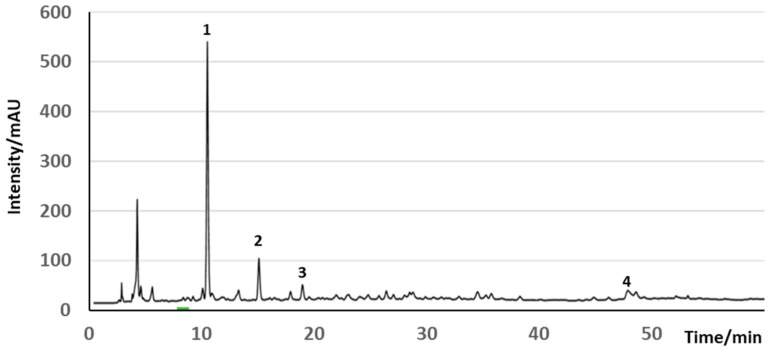
HPLC chromatogram of an olive pomace extract with detection at 280 nm. 1, hydroxytyrosol; 2, tyrosol; 3, caffeic acid; 4, conselogoside.

In addition to olive oil, OP contains highly valuable compounds, such as lignocellulose, an insoluble dietary fiber, minerals, such as potassium, phenolic compounds [40,41,42], and tocopherols [37,43,44,45]. The high lignin content, however, makes its direct use in food challenging. Nevertheless, the most important bioactive compounds found in OP in concentrations around 2 g/kg are phenolic compounds, which are not only responsible for the OP’s phytotoxicity but also its health and well-being applications in foods and dermatological products [32,46]. The major phenols in OP are HT, representing more than 55% of the polyphenolic content of the OP extracts, tyrosol and conselogoside (up to 25%) (Figure 6) [20], but an important number of other polyphenols can be observed in many OP extracts. Further phenolic compounds found in OP in smaller concentrations are HT glycosides, caffeic, *p*-coumaric and ferulic acids, verbascoside, oleuropein and demethyloleuropein, ligstroside, oleacein, OC, verbascoside, and luteolin-O-rutinoside [26,45,47,48]. The contribution of these compounds to human health is now well documented in the literature [26,45,47,48].

However, because of the oxidative instability and occurrence of hydrolysis due to the presence of enzymes and water, the polyphenol content changes over time, both in quantity and quality. Nevertheless, the activity of endogenous hydrolases may represent an opportunity to obtain a consistent product, rich only in HT.

Recently, a simple pilot-scale process was carried out for the recovery of concentrated aqueous solutions of hydroxytyrosol and tyrosol from olive oil pomace [49]. The process consisted of performing an aqueous extraction from olive pomace, followed by a nanofiltration step and a final concentration using reverse osmosis. The results obtained showed that the final concentration in the reverse osmosis retentates depends directly on the concentration of free hydroxytyrosol and tyrosol in the nanofiltration feed. The concentration of both compounds varied considerably between harvest seasons and with storage time, attaining a maximum value approximately 5 months after olive oil production. The concentration of these produced concentrated solutions remained stable for at least 14 months [49]. This kind of product has several applications, such as for ingredients in the food, cosmetic, pharmaceutical, and nutraceutical production industries.

#### 3.2.2. Olive Mill Wastewater

Olive mill wastewater (OMWW) is a by-product generated from the combination of water contained in fruits with the water added during the fruit washing and extraction process. It can be collected from different stages of the olive oil production process, namely during separations carried out either by horizontal (decanters) or vertical centrifuges [48,50,51].

It consists mainly of water (83–92%) and has a high concentration of phytotoxic hydrophilic compounds (up to 16%), including tannins and phenolic compounds [32,52,53]. The dispersion of this wastewater into the environment, without prior treatment, is a major environmental problem as it causes a high consumption of oxygen, as well as changes in the color and odor of natural waters, and is, therefore, toxic to aquatic organisms (the lethal dose for fish is of 8%), leading to changes in the quality of soils [53,54,55].

Around 50 phenolic compounds have already been identified in OMWW [6,53,56,57]. However, their concentration of compounds varies significantly as it depends on the initial phenolic compound composition of the olive from which it originates, the extraction methods used, and the conditions and time of storage of this by-product [53,56,57]. Similar to OP, the phenol that stands out in the highest concentration is HT [38,55,58,59,60,61,62]. However, it is still possible to observe the presence of other water-soluble phenolic compounds, such as hydroxycinnamic acids, namely caffeic, *p*-coumaric and ferulic acids, gallic acid, various flavonoids, such as luteolin, and its glycosidic derivatives, oleuropein, desmethyloleuropein, isoacteoside, and ligstroside. Usually, its oleuropein content is not very high due to the hydrolytic degradation that it undergoes during the olive oil extraction process [12,32,33,60]. Given the water solubility of these phenolic compounds, they can be separated and concentrated using low-cost membrane technologies [63]. Once again, the biggest problem with this raw material is its low chemical stability caused by both hydrolysis and oxidation. The oxidation of phenols produces quinones and polymeric phenols with a structure similar to that observed for lignin, and gives this biowaste a similar color to that of OP, from dark red to black [50,53,59].

Given its toxicity, it is necessary to carry out prior treatment of this waste, which is generally divided into two stages: the first stage usually involves evaporation in tanks [55,56,60] and the second stage involves various physical, chemical, and biological treatments, which include decantation, sedimentation, and coagulation followed by anaerobic digestion [55,56,60]. This process is normally very time-consuming, attracts insects, and can contaminate groundwater if the tank is not watertight [32]. During this process, many phenolic compounds degrade, mainly through oxidation.

#### 3.2.3. Olive Leaves

Both leaves and branches are used as biomass in energy production or crushed to be used in ruminant animal feed [48,64]. Within this biomass, around 50% of the weight is generated from fine branches, 25% is generated from the leaves, and the remaining 25% is made up of coarse branches or wood [61]. However, an alternative to this by-product, in particular the leaves portion, is its use as a raw material for the development of new products with high added value, which may be used in the pharmaceutical, cosmetic, and food industries given their high content of phenolic compounds, oil, protein, fiber, and other potentially valuable secondary metabolites [48,64]. Traditional medicine has long used leaf infusions for the treatment of diabetes, gout, fever, rheumatism, and hypertension, as well as for the prevention of CVD. Therefore, leaves have been valued for their health benefits and can be used in infusions or to obtain dry extracts sold in tablets and capsules as supplements, with diuretic, hypotensive, anti-inflammatory, and antioxidant effects [49,65]. Olive leaf is also considered a safe and natural remedy to support a healthy immune defense system. From the several studies that have been carried out, essentially in vitro, the bioactivity of dry extracts and infusions of olive leaves has been essentially related to their high polyphenol content, in particular of oleuropein, although which polyphenols present the various bioactivities is not yet fully established [66,67,68]. Data reported to date are scare but generally show the consistent and health-enhancing properties of leaf extracts, but knowledge remains limited, particularly in clinical trials, with little being known about the underlying mechanisms of action, dose–response relationship, and long-term impact [67,68]. It is important to note that international regulatory agencies, like the Food and Drug Administration, do not regulate nutritional supplements, like olive leaf extract. However, because olive leaves have been an important part of the Mediterranean diet for centuries, the extract is being commercialized and considered safe for most people, with a potential application in the field of innovative functional foods [69]. Additionally, their application has also been studied in the cosmetic and dermatology research field for the treatment of skin diseases and aging using different topical delivery systems [70,71,72].

Unlike other olive by-products, leaves can be obtained throughout the year, although their availability is higher during the olive tree pruning periods and during olive oil production, where they can represent around 10% of the material that arrives at the mill [20]. Olive leaves contain about 30–40% lignin, 9% crude protein, 5–7% cellulose, and 3–4% hemicellulose, and may also contain up to 2–3% of phenolic compounds [64]. However, as it is a living plant material, its composition in terms of various compounds varies with the same factors that affect the composition of olives, namely soil and climate conditions, time of year, cultivars, storage conditions, and extraction methods [73].

Phenolic compounds are found in high concentrations in leaves as they provide a natural defense against predators [47]. The phenols normally present in higher concentrations in leaves are the bitter glycoside oleuropein and its secoiridoid derivatives [26,47,74]. Thus, the leaves, unlike what happens with OP and wastewater, present the most interesting by-product for the extraction of secoiridoids and the preparation of phenolic extracts with high added value. In addition to these compounds, lignans (such as pinoresinol or 1-acetoxypinoresinol), other secoiridoids (such as ligstroside and its demethylated derivative), phenolic alcohols (HT), and several flavonoids and flavonols, cinnamic acid derivatives (such as verbascoside), and phenolic acids can also be found [26,47,48,69,73,74,75,76,77,78]. Oleuropein and its derivatives have an important antioxidant and antimicrobial capacity, rendering them as good alternatives to synthetic preservatives [79,80]. The phenolic composition of leaf extracts can be variable and, therefore, they may have several biological activities, acting as antioxidant, anti-inflammatory, antibacterial, and anticancer agents [14,81].

**Figure 7:**
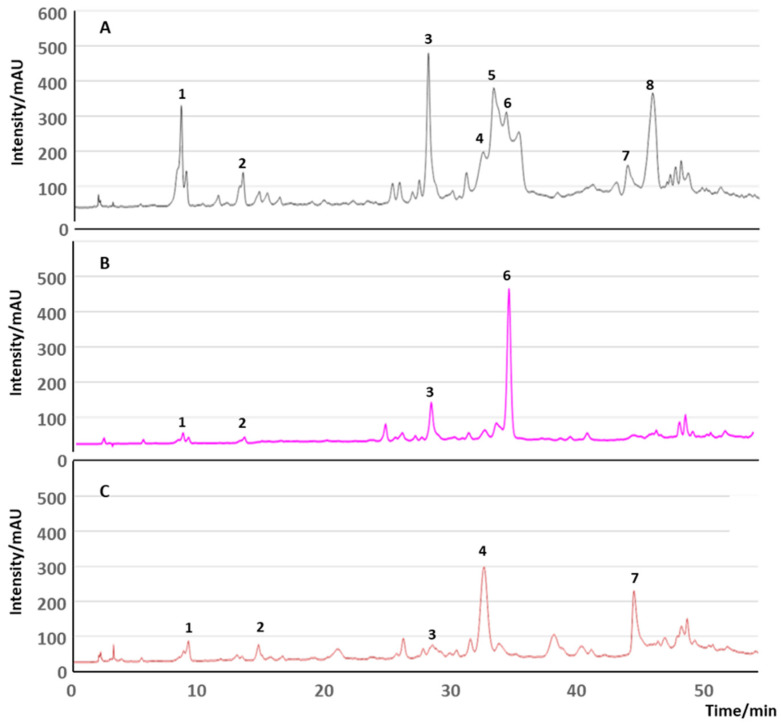
HPLC chromatogram of olive leaf extracts with detection at 280 nm. Leaves were extracted by maceration in methanol after storage under different conditions: (**A**)—fresh leaves; (**B**)—leaves allowed to dehydrate at 38 °C for 48 h (kept in an open box); (**C**)—leaves not allowed to dehydrate stored at 38 °C for 18 h (kept under vacuum in a plastic bag). 1, hydroxytyrosol; 2, tyrosol; 3, ferulic acid; 4, oleacein; 5, oleuropein aglycone derivative; 6, oleuropein; 7, caffeoyl-6-secologanoside; 8, oleuropeidine. Data from [83].

An interesting feature of this by-product is the possibility of modulating the phenol content of leaves by thermal treatments prior to extraction (Figure 7) [19]. Leaves can actually be enriched in oleuropein or oleacein depending on this treatment [82]. The storage of leaves at 38 °C can either increase the oleuropein content, when leaves are dehydrated, or reduce the total amount of phenols with a change in the phenolic composition of the leaf extract, so it is closer to the composition of VOO extract, when leaves are not allowed to dehydrate [82].

### 3.3. Extraction Methods

Biowaste from the olive oil industry can be an important source of bioactive phenolic compounds. Currently, one of the greatest difficulties in obtaining phenolic compounds extracts and their subsequent use is related to extractive methods. Therefore, to obtain good yields in this process, avoiding structural changes or co-elution of undesirable compounds from plant matrixes, such as chlorophylls, it is necessary to select one or more suitable solvents for the substances of interest and perform multistep extractions [84]. Additionally, it is necessary to optimize pH, temperature, and time of extraction among other factors [84].

As the phenolic compounds found in olive by-products have a certain range of polarity, it is possible to use various solvents to produce extracts richer in certain compounds. Usually, the extraction of the simplest, water-soluble phenols, such as HT, is favored by water or water/generally recognized as safe (GRAS) organic solvent mixtures containing high percentages of water, but the more lipophilic compounds, such as oleacein, OC and oleuropeidin, are preferably extracted with ethanol or mixtures containing higher percentages of this solvent [11,12,20,21,85]. Therefore, the extraction process must consider the type and characteristics of the residues and plant materials included in the sample and the target compounds to be extracted. Recently, some studies have been reported on the application of natural deep eutectic solvent (NaDES)-based extraction for the recovery of polyphenols from vegetal by-products, including OP [86,87,88]. NaDES are environmentally friendly solvents made up of mixtures of choline chloride or betaine and various sugars, namely fructose and glucose, or edible acids, such as lactic acid and citric acid. These solvents have considerable potential for extracting bioactive compounds and, depending on the application, can be incorporated into the final product as ingredients without the need to eliminate the solvent.

The biggest challenge in using industrial waste of vegetable origin to produce standardized extracts with high added value for different applications lays in the fact that these wastes are obtained from raw materials with different characteristics and are not controllable by the company receiving the agro-industrial waste. In the case of the olive oil industry, the olive cultivar and state of maturation, as well as the methods of extraction and conservation of residues, can vary greatly, resulting in by-products with different compositions, both quantitatively and qualitatively. Therefore, the choice of the method of conservation and the pre-treatment applied to the material to be extracted can be made considering the purpose of the extract and the ability to produce an extract with the most standardized composition possible.

The extraction of olive tree polyphenols from residues and by-products can be carried out by both conventional and innovative methods. Conventional methods include maceration and Soxhlet extraction. The major problem arising from these methodologies is that usually the use of large volumes of solvents and high temperatures has the potential of originating ecological problems and the degradation of thermolabile compounds [89]. Innovative methods aim for a highly efficient recovery of compounds employing green technologies and solvents [90]. Currently, there are several green extraction methodologies, namely, microwave-assisted extraction, ultrasound-assisted extraction, pressurized liquid extraction, high-voltage electrical discharge, pulsed electric field, and supercritical fluid extraction [91]. Application of these emerging extraction technologies allows for low energy costs and avoids, in many cases, the degradation of bioactive compounds due to the higher temperatures and longer extraction times required, thus avoiding the rapid degradation of many phenols [92]. In the literature, many references report the application of these methods to olive oil by-products, including the leaves, OP, and OMWW [88,93,94,95,96,97,98,99,100,101,102,103,104,105].

## 4. Polyphenols and Alzheimer’s Disease

### 4.1. Overview of Alzheimer’s Disease

AD is the most common neurodegenerative disorder and the leading cause of dementia worldwide, affecting millions of people [106]. Characterized by progressive cognitive decline and memory loss, AD primarily affects episodic memory in its initial phases and later leads to severe cognitive impairment and loss of independence [107]. The pathological hallmarks of AD include extracellular deposition of amyloid-beta (A*β*) plaques and intracellular accumulation of neurofibrillary tangles (NFTs) composed of hyperphosphorylated tau protein. A*β* plaques form when the A*β* precursor protein (APP) is cleaved by beta and gamma-secretases, resulting in the release of A*β* peptides. These peptides aggregate into insoluble plaques, which disrupt cell-to-cell communication and activate immune responses, leading to inflammation and neuronal damage [108,109,110]. The areas which include not only deposits of amyloid throughout the cortex and hippocampus of the brain, but also dystrophic neurites and activated microglia, which indicate neuroinflammation, are commonly referred to as neuritic plaques [111]. On the other hand, NFTs occur due to abnormal hyperphosphorylation of tau protein, which normally stabilizes the microtubules. Hyperphosphorylated tau detaches from the microtubules, forming insoluble tangles that interfere with neuronal transport and contribute to cell death [110,112]. Progressive deposition of A*β* protein is linked to neurodegenerative changes, including synaptic [113,114] and neuronal loss [115,116], microglial cell proliferation [117,118], and astrogliosis [119,120]. Recent studies have suggested that neurodegeneration in AD may also involve interference with adult hippocampal neurogenesis, with significant abnormalities observed in transgenic animal models of the disease [121,122]. Cognitive changes in AD are primarily associated with synaptic injury in the limbic system and the neocortex [123].

The etiology of AD is complex and involves genetic [124], immunological [125,126], and environmental [127,128,129,130] factors that disrupt homeostatic mechanisms in the brain. Most AD cases occur sporadically, but certain susceptibility genes, such as apolipoprotein E (APOE), can increase the risk. The APOE ε4 allele remarkably increases the risk of AD, whereas the ε2 allele offers relative protection compared to the common ε3 allele [131,132]. Familial AD, which represents the minority of cases, is caused by mutations in genes encoding APP, presenilin 1 (PS1), or presenilin 2 (PS2) [133,134,135]. Efforts to develop effective therapies for AD have focused on the removal of A*β* from the brain. Understanding the mechanisms of A*β* clearance, including proteolytic enzymes and chaperone molecules, such as APOE, is essential for these therapeutic strategies [136]. The heterogeneous nature of AD, involving various pathological processes, such as oxidative stress [137,138], mitochondrial dysfunction [139], autophagy [140,141], noncoding RNAs [142], and neuroinflammation, highlights the complexity of the disease and the multifaceted approaches required to address it.

#### 4.1.1. Oxidative Stress

Oxidative stress is a critical factor in AD development and progression. The brain’s high oxygen consumption [143], abundant lipid content [144,145], and relatively low levels of antioxidant defenses [145] render it particularly vulnerable to oxidative damage. In AD, oxidative stress arises from the overproduction of reactive oxygen species (ROS) and reactive nitrogen species, which harm cellular components including DNA, proteins, and lipids [146]. Elevated levels of 8-hydroxy-2′-deoxyguanosine indicate DNA oxidative damage [147], while increased malondialdehyde (MDA) levels indicate lipid peroxidation in AD brains [148]. Dysregulation of metals ions, such as iron, copper, and zinc, is observed in AD. These metals can catalyze ROS production via Fenton’s reactions, thereby increasing oxidative stress. Iron accumulates in AD brains, especially in areas with amyloid plaques and NFTs, further contributing to oxidative damage. Oxidative stress appears early in AD, even before the onset of symptoms and A*β* plaque formation, indicating its potential causative role [149]. Mitochondrial abnormalities are prevalent in AD brains, including increased oxidative damage to the mitochondria [150], reduced mitochondrial DNA [151], and impaired mitochondrial function [150,151]. These abnormalities correlate with heightened oxidative stress and neurodegeneration.

#### 4.1.2. Autophagy

Autophagy, a cellular process for degrading and recycling damaged components, is essential for maintaining neuronal homeostasis. In AD, mitochondrial dysfunction and oxidative stress impair autophagy, leading to accumulation of damaged mitochondria and other cellular debris. This build-up exacerbates oxidative stress and neuronal dysfunction [152,153]. The interplay between mitochondrial dysfunction and autophagy in AD involves several mechanisms. The mitochondria may undergo selective degradation (mitophagy) to remove damaged organelles. However, during aging and AD progression, this process becomes inefficient, leading to the accumulation of lipofuscin and other non-degradable materials in the lysosomes. This accumulation hampers the ability of cells to degrade and recycle damaged components, further promoting oxidative damage and neurodegeneration. Therapeutic interventions targeting mitochondrial dysfunction and enhancing autophagy have potential for AD treatment [152,153]. Understanding the specific roles of mitochondrial dysfunction and autophagy at different stages of AD progression is crucial for the development of effective therapies.

#### 4.1.3. Chronic Neuroinflammation

Chronic neuroinflammation is a significant feature of AD, in which activated microglia and astrocytes release pro-inflammatory cytokines and chemokines that can exacerbate neuronal injury and promote A*β* and tau pathology. This inflammation and microglial activation contribute to neuronal damage and the spread of pathology, emphasizing the role of glial cells, especially astrocytes, in AD. Astrocytes maintain brain homeostasis by providing metabolic support to neurons, recycling neurotransmitters, stimulating synaptogenesis, forming part of the blood–brain barrier (BBB), and regulating blood flow [154]. Emerging evidence indicates that many genetic risk factors for late-onset AD, such as APOE, clusterin, and sortilin-related receptor genes, are predominantly expressed in glial cells, including astrocytes, microglia, and oligodendrocytes. This shift in focus from neurons to glial cells, particularly microglia and neuroinflammation, is significant for AD research [155]. Recent rodent studies suggest that astrocytes, which undergo morphological and functional changes, contribute directly to neuroinflammatory and neurodegenerative processes in AD. Reactive astrocytes near amyloid plaques show spontaneous calcium oscillations and abnormal intercellular calcium waves, contributing to neuroinflammatory processes through the upregulation of inflammatory genes [156]. These reactive astrocytes are distinct from atrophic astrocytes, which exhibit reduced volume and process loss, and likely lose their homeostatic functions. Astrocytes also play a role in A*β* clearance by expressing enzymes and proteins involved in A*β* metabolism and transport across the BBB [156].

Moreover, the major genetic risk factor APOE4 influences A*β* uptake and plaque formation. APOE4 astrocytes exhibit impaired A*β* uptake and cholesterol accumulation, which are related to defective autophagy and endosomal acidification [157,158]. In AD, astrocytes exhibit both neurotoxic and neuroprotective functions. A1 neurotoxic astrocytes, induced by activated microglia-releasing factors, such as interleukin-1 alpha (IL-1*α*), tumor necrosis factor alpha (TNF*α*), and complement component 1q, express complement components, such as C3, contributing to neuronal death and synaptic dysfunction. Conversely, A2 astrocytes, which are induced by ischemia, upregulate neurotrophic genes and secrete factors that promote neuronal survival and synaptic repair [159]. The dual role of astrocytes in AD highlights the need for a deeper understanding of their various states during disease progression.

#### 4.1.4. Gut Microbiota

In recent years, research has highlighted the role of the gut microbiota in AD progression. Changes in the composition of the intestinal microbiota have been closely linked to AD, suggesting a critical role in the onset and progression of the disease. A bidirectional relationship exists between the gut microbiota and the brain, known as the microbiota–gut–brain axis [160]. Experimental studies indicate that the gut flora plays a role in brain functions, such as memory and learning, and its function and composition affect age-related cognitive impairment and dementia. The interaction between the gut flora and central nervous system occurs through the nervous system or chemicals that cross the BBB [160]. The gut microbiota produces substances that influence neuronal activity and behavior, while the brain can affect the gut microbiota through neurotransmitter signals. There are also communication pathways between the brain and gut, with the vagus nerve playing a central role, linking the gut and the autonomic nervous system [160,161]. The enteric nervous system also sends and receives signals from the central nervous system via the gut flora. Additionally, blood circulation facilitates exchanges between the gut and brain, with the intestinal mucosa and BBB allowing the passage of endocrine and immune molecules, affecting both gut and brain functions [160].

#### 4.1.5. Synaptic Hypothesis in AD

The synaptic hypothesis of AD emphasizes the role of synaptic dysfunction and loss in disease progression. Extensive research supports this hypothesis, revealing that many key changes in AD occur on the postsynaptic side of the synapse, particularly in dendrites. The dendritic hypothesis builds on this idea, suggesting that processes occurring in and around neuronal dendrites are central to the pathogenesis of AD. Dendritic abnormalities in AD are widespread and manifest early in the disease’s course [162]. These abnormalities include dystrophic neurites [163], reduced dendritic complexity [164], and the loss of dendritic spines [165]. Dystrophic neurites often associated with amyloid plaques indicate a significant dendritic pathology [163]. Reduced dendritic complexity, characterized by decreased branching and connectivity, particularly in regions, such as the hippocampus, is linked to NFTs and loss of afferent inputs [164]. The loss of dendritic spines, which are crucial for synaptic connections, contributes to the synaptic dysfunction observed in AD [165]. A*β* exerts toxic effects on dendrites by binding to dendritic receptors and disrupting the normal ion channel function. Moreover, tau is mislocalized in dendrites, disrupting microtubule stability and contributing to synaptic dysfunction. The interaction between A*β*, tau, and genetic factors creates a cascade of pathological events that leads to dendritic dysfunction and neuronal loss [162]. Understanding these mechanisms is crucial for the development of targeted therapies aimed at preserving dendritic integrity and preventing AD progression.

### 4.2. Preclinical Models for Assessing Polyphenol Therapeutics in Alzheimer’s Disease

Research into the mechanism of development of AD is fundamental, as it represents a significant health challenge. Postmortem patient brains provide an essential starting point for understanding the effects of AD and neurodegeneration in the brain. Nonetheless, this approach is limited by the inability to evaluate potential treatments or observe how interventions might alter the progression of the disease. Hence, the use of cultured human cells is crucial [166]. When selecting cell lines to study the effects of polyphenols on AD, scientists use a variety of models, each providing unique advantages and valuable insights.

#### 4.2.1. Induced Pluripotent Stem Cells (iPSC)-Derived Neurons

Induced pluripotent stem cells (iPSC)-derived neurons are valuable for studying human-specific pathways and genetic factors, whereas primary neuronal cultures provide a more physiologically relevant environment to explore disease mechanisms and drug effects. iPSC-derived neurons can be generated by gene editing cells from both healthy individuals and AD patients. These cells can then be used to create 3D in vitro human brain models that may have a functional BBB, constituting a sophisticated model [166] of neurodegenerative processes of AD, allowing for the investigation of disease mechanisms and identification of potential therapeutic targets (Table 1) [167,168]. Interestingly, studies have shown that the differentiation and maturation of iPSC-derived neurons can be optimized to produce mature neurons more rapidly, which makes them suitable for high-throughput screening [168]. This is particularly relevant for AD research, in which the ability to screen for drugs that may modify disease pathways is crucial. Additionally, the role of histone deacetylase 2 (HDAC2) in the differentiation and maturation of iPSC-derived neurons has been highlighted, with implications for neurodegeneration and brain aging, both of which are central to AD pathology [167].

#### 4.2.2. Primary Neuronal Cells

Primary neuronal cultures facilitate the genetic manipulation of neurons, time-lapse imaging, and drug screening, which are essential for understanding AD mechanisms and evaluating potential therapies (Table 1) [169]. Moreover, they allow for the validation of genes associated with AD, such as those modulating A*β* peptide levels, which is critical for the development of disease-modifying treatments [170]. However, there are limitations to the use of primary neuronal cultures. They may not fully recapitulate the complex architecture and cell-to-cell interactions of the brain, which are important for understanding the progression of neurodegenerative diseases, like AD (Table 1) [171]. Additionally, while primary neuronal cultures are valuable for studying apoptosis and its regulation, which is implicated in AD and other neurodegenerative disorders, they do not encompass all aspects of the disease (Table 1) [172]. Future research should employ multiple cell lines to capture the full spectrum of polyphenolic effects. Employing multiple cell lines is essential to understand the multifaceted effects of polyphenols on AD. This approach allows the evaluation of the impact of polyphenols on various pathological aspects of AD and addresses the challenges posed by their complex pharmacokinetics and bioavailability [173]. The use of a spectrum of cell lines can aid in identifying the most effective polyphenolic compounds and optimizing their therapeutic application in AD (Table 1) [173,174]. This approach allows for the cross-validation of the results and a more comprehensive understanding of the mechanisms involved. Given the complexity of AD and the multifaceted nature of polyphenols, combining different models could lead to more robust and generalizable results.

#### 4.2.3. APP/PS1 Mice

Various animal models have been used to investigate the therapeutic effects of polyphenols against AD, each of which offers unique advantages and limitations. One of the most used models is a transgenic mouse model, specifically APP/PS1 mice. These mice express mutated human APP and PS1, leading to A*β* plaque formation similar to that in human AD. The main advantage of this model is the rapid and robust development of A*β* pathology, making it a reliable model for studying the effects of polyphenols on amyloid deposition (Table 2 and Table 3) [175,176]. However, a significant disadvantage is that APP/PS1 mice do not fully replicate the tau pathology observed in human AD. This limitation means that, while they are useful for studying A*β*-related aspects of AD, they may not provide a complete picture of the disease’s progression and pathology, particularly in terms of NFTs and some behavioral changes [176].

#### 4.2.4. APPSwe/PS1dE9 Mouse

Another commonly used transgenic model is the APPSwe/PS1dE9 mouse (Table 2 and Table 3). The advantages of this model include its ability to demonstrate significant reductions in brain and serum A*β* levels, amyloid plaques, and inflammatory markers. However, the model shares limitations with APP/PS1 mice in not fully capturing tau pathology [177,178].

#### 4.2.5. Senescence-Accelerated Mouse Prone 8 (SAMP8)

The SAMP8 model is another important animal model used in AD research (Table 2 and Table 3). SAMP8 mice exhibit accelerated aging and develop AD-like symptoms, including increased levels of A*β* peptides and tau hyperphosphorylation. The main advantage of the SAMP8 model is its ability to mimic the aging process, a major risk factor for AD. However, a disadvantage is that the accelerated aging might not fully represent the typical onset and progression of human AD, which generally develops over a much longer period [179,180].

#### 4.2.6. Sprague–Dawley Rats

Sprague–Dawley rats have been used to demonstrate the potential therapeutic effects of polyphenols in modulating AD pathogenesis. The advantage of using rats is their large brain size, which facilitates certain types of experimental procedures and brain analyses. However, the major disadvantage is that rat models do not naturally develop AD-like pathology and require artificial induction, which may not fully replicate human conditions [181].

#### 4.2.7. Caenorhabditis Elegans

Invertebrate models, such as *C. elegans*, have also been used to study the effects of polyphenols (Table 2 and Table 3). These models offer advantages, such as ease of genetic manipulation, short lifespan, and low cost, making them useful for high-throughput screening of potential therapeutics. However, a significant limitation is that the simplicity of the nervous system does not capture the complexity of the human brain and the AD pathology [182].

#### 4.2.8. Drosophila Melanogaster

*D. melanogaster* (fruit fly) is another valuable model for AD research (Table 2 and Table 3). These flies can be genetically modified to express human A*β* or tau proteins, leading to AD-like symptoms, such as amyloid plaques and neurodegeneration. The advantages of using Drosophila include its short life cycle, ease of genetic manipulation, and cost-effectiveness, which allows for the rapid screening of numerous compounds. Drosophila models have also been used to evaluate the effects of various polyphenols. However, a significant disadvantage is that despite their utility in high-throughput screening and initial functional studies, the simplicity of their nervous system and differences in physiology from mammals can limit the direct applicability of these findings to human AD [183]. Consequently, the choice of animal model depends on the specific aspects of the AD being studied and the type of polyphenols being tested.

**Table 1:** In vitro studies on polyphenols: mechanisms of action in Alzheimer’s disease prevention.

Polyphenol	Chemical Formula	Plant Origin	Experimental Model	Health Effects	Reference
**Flavonoids**					
Apigenin	C_15_H_10_O_5_	Some of the primary sources of apigenin include: Parsley (*Petroselinum crispum*) Celery (*Apium graveolens*) Chamomile (*Matricaria chamomilla*) Thyme (*Thymus vulgaris*) Peppermint (*Mentha piperita*) Oregano (*Origanum vulgare*) Onions (*Allium cepa*) Oranges (*Citrus sinensis*) Artichokes (*Cynara scolymus*)	Human-induced pluripotent stem cell (iPSC)-derived neurons. For the inflammation-based assays, neurons were treated with 50 μM apigenin or a vehicle control for 24 h. Following this treatment, the media was replaced with conditioned media from activated microglia for 48 h. In addition to the inflammation assays, the neurons were subjected to oxidative stress by treatment with 300 μM H_2_O_2_ or 10 μM SNAP for 24 h, after a 24 h pre-treatment with 50 μM apigenin.	Apigenin: Significant anti-inflammatory and neuroprotective effects. Protects neurons from neurite retraction induced by inflammatory stress. Reduces the release of NO and various pro-inflammatory cytokines. Protects neurons from apoptosis by reducing caspase-3/7 activity. Reduces neuronal hyper-excitability and disturbances in calcium signaling. Decreases both oxidative and nitrosative stress levels.	[184]
Cyanidin-3-O-glucoside Malvidin-3-O-glucoside Pelargonidin-3-O-glucoside Peonidin	Cyanidin-3-O-glucoside: C_21_H_21_O_11_ Malvidin-3-O-glucoside: C_23_H_25_O_12_ Pelargonidin-3-O-glucoside: C_21_H_21_O_10_ Peonidin chloride (Peonidin): C_16_H_13_ClO_6_		SK-N-SH cell line (human neuroblastoma cell line). The polyphenol formulation tested was MAF14001, an equimolar mixture of cyanidin-3-O-glucoside chloride, malvidin-3-O-glucoside chloride, pelargonidin-3-O-glucoside chloride, and peonidin chloride. This formulation was tested at concentrations ranging from 5 to 20 µM. The administration mode involved direct addition of MAF14001 to the cell culture medium. The cells were incubated with the formulation for 24 h to assess the protective effects against A*β* peptide-induced toxicity.	MAF14001, a mixture of anthocyanins and anthocyanidins: Protects SK-N-SH cells against A*β*-induced toxicity. Prevents A*β*-induced oxidative stress, mitochondrial dysfunction, and apoptosis Interacts with A*β* to prevent its aggregation, a key process in A*β*-induced oxidative stress. Decreases TAU phosphorylation induced by A*β*.	[185]
Epicatechin (EC) Catechin (CE)		*Theobroma cacao*	Mouse hippocampal cell lines HT-22. Primary neuronal cultures. The cells were treated with A*β* oligomers (12.5 µM) for 24 h to mimic AD. Differentiated cells were treated with cocoa extract containing 30 µg/mL EC, 10 µg/mL CE, and 170 µg/mL total polyphenols for 4 days.	Cocoa polyphenolic extract: Exerts neuroprotective effects by activating the BDNF survival pathway. This activation counteracted neurite dystrophy induced by A*β* plaques and oligomers, suggesting the potential of cocoa polyphenols as preventive agents against neurodegenerative diseases, such as AD by reducing oxidative stress and promoting neuronal survival.	[186]
Epicatechin (EC) Catechin (CE) Procyanidins	EC: C_15_H_14_O_6_ CE: C_15_H_14_O_6_ Procyanidins: Varies depending on the degree of polymerization (DP)	*Theobroma cacao*	Mouse brain hippocampal slices. Slices were exposed to 25 µM and 100 µM concentrations of cocoa extracts. The hippocampal slices were initially acclimated in oxygenated artificial cerebrospinal fluid and subsequently incubated with the cocoa extracts at 32 °C for a duration of 1 h.	Cocoa polyphenolic extract: Reduces A*β* oligomer formation. Prevents synaptic deficits induced by A*β* oligomers. Restores long-term potentiation reduced by oligomeric A*β*.	
Epigallocatechin-3-gallate (EGCG)	C_22_H_18_O_11_	*Camellia sinensis*	Human SH-SY5Y neuroblastoma cells and Chinese hamster ovary. Cells transfected with human APP695 were treated with 1–10 μM EGCG for 2 days.	EGCG: Demonstrates potent iron-chelating activity comparable to that of desferrioxamine. Significantly reduces both APP and toxic A*β* peptide production Promotes non-amyloidogenic APP processing through increased secretion of soluble APP-*α* and activation of PKC.	[187]
Epigallocatechin-3-gallate (EGCG)	C_22_H_18_O_11_	*Camellia sinensis*	Cholinergic-like neurons (ChLNs) derived from umbilical cord mesenchymal stem cells with either wild-type or PSEN1 E280A mutation. The cells were exposed to varying concentrations of EGCG, ranging from 5 to 50 µM, with 50 µM selected for further experiments based on its efficacy in previous tests. The EGCG was administered directly into the regular culture medium for a duration of 4 days post-transdifferentiation.	EGCG: Inhibits APP aggregation. Blocks the phosphorylation of TAU (p-TAU), increases mitochondrial membrane potential (∆Ψm), and decreases oxidation of DJ-1 at the residual Cys106-SH. Inhibits the activation of transcription factors c-JUN and P53, as well as PUMA and CASPASE-3, in mutant ChLNs compared to WT. Reverses Ca^2+^ influx dysregulation in response to ACh stimuli in PSEN1 E280A ChLNs. Inhibits the activation of the transcription factor NF-κB and reduces the secretion of the pro-inflammatory cytokine IL-6 in wild-type astrocyte-like cells when exposed to the culture supernatant from mutant ChLNs.	[188]
Luteolin (LUT)	C_15_H_10_O_6_	Various plants including parsley, thyme, and celery	Human neuroblastoma BE(2)-M17 cells Cells were pre-treated DHA, LUT, and urolithin A (UA), each at a concentration of 5 µM in combination (D5L5U5) and individually at 30 µM, 20 µM, and 30 µM respectively. The pre-treatment lasted for 24 h, after which the cells were exposed to 20 µM of oligomeric A*β*1–42 for various durations ranging from 4 to 72 h	DHA, LUT, and UA in combination (D5L5U5): Have potent inhibitory effects against A*β*1–42-induced toxicity through protecting mitochondria. These effects include minimizing oxidative stress, increasing ATP levels, and inducing mitophagy and mitobiogenesis.	[189]
Quercetin Thymol	Quercetin: C_15_H_10_O_7_ Thymol: C_10_H_14_O	Quercetin: found in *Allium cepa*, asparagus, red leaf lettuce, apple, and berries. Thymol: major component of T*hymus vulgaris.*	PC12 cell model The concentrations of the polyphenols used were 40 µM for quercetin and 35 µg/mL for thymol, while the concentrations of Allium cepa extract were 1000 µg/mL and Thymus vulgaris essential oil was 0.4 ppm. The incubation period for these treatments was 48 h. The cell model was created using formaldehyde at a final concentration of 0.35 mM to induce AD-like conditions in the PC12 cells.	Quercetin: Reduces the rate of apoptosis in AD cells significantly better than other compounds. Increases the expression of genes related to AD, such as PP2A, GSK3, and NMDAR. Thymol: Shows anti-AD and antioxidant effects, increases cell survival rate, and reduces apoptosis rate. Both thymol and Thymus vulgaris essential oil significantly increase the expression of the PP2A gene.	[190]
**Phlorotannins**					
Eckol, 6,6′-Bieckol, 8,8′-Bieckol Dieckol Phlorofucofuroeckol-A (PFF-A)	Eckol: C_18_H_12_O_9_ 6,6′-Bieckol and 8,8′-Bieckol: C_36_H_22_O_20_ Dieckol: C_36_H_22_O_18_ Phlorofucofuroeckol-A: C_30_H_18_O_13_	*Ecklonia cava*	SK-N-SH neuroblastoma cells. Cells were exposed to 2 μL of 50 mM of each polyphenol for 24 h.	*Ecklonia cava* extract: Inhibits AChE activity (IC50 from 16.0 to 96.3 μM). Shows a potent BuChE inhibitory activity, particularly phlorofucofuroeckol-A (PFF-A) with an IC50 of 0.95 μM. Inhibits GSK-3*β*, which is involved in the formation of hyper p-TAU and generation of A*β*. Bieckol and PFF-A inhibit APP biosynthesis. PFF-A exhibits strong *β*-BACE-1 inhibitory activity.	[191]
**Phenylpropanoids, Flavonoids, and Hydroxycinnamic acids**					
Phenylpropanoids Flavonoids Hydroxycinnamic acids		*Arabidopsis thaliana* mutants, including prn1, cop1, and xpf3	Primary mixed glial cultures derived from the cerebral cortex of neonatal APOE-targeted-replacement (APOE-TR) and APOE-knock-out (KO) mice. These cultures contained approximately 95% astrocytes and 5% microglia. Cells were treated with the plant extracts at a concentration of 150 µg/mL, either with or without inflammatory stimuli (LPS or A*β* oligomers). Incubation times included pre-treatment periods followed by inflammatory stimulation, with TNF*α* levels measured after 16 h.	Polyphenol-enriched extracts from *Arabidopsis thaliana* mutants: Exhibits anti-inflammatory effects, especially the xpf3 mutant. Attenuates TNF-*α* secretion in mixed glial cultures, with a notable reduction observed in APOE4 cultures compared to APOE3 and APOE2. Could serve as a potential therapeutic agent for AP-OE-modulated neuroinflammation, a characteristic of AD.	[192]
**Stilbenes**					
Resveratrol	C_14_H_12_O_3_	Grapes and red wine	HEK293 cells stably transfected with human APP695. N2a cells stably transfected with wild-type or Swedish mutant human APP695 cDNAs. Cells were treated with 20–40 µM resveratrol for 24 h or 10 or 20 µM resveratrol for 12, 48, and 72 h.	Resveratrol: Decreases levels of secreted and intracellular A*β* peptides in the cell lines. Does not inhibit A*β* production but promotes intracellular degradation of A*β* via a mechanism involving proteasome involvement. Decreases in A*β* could be prevented by selective proteasome inhibitors and by siRNA-directed silencing of the proteasome subunit *β*5. Demonstrates a proteasome-dependent anti-amyloidogenic activity, suggesting therapeutic potential in AD.	[193]
Resveratrol	C_14_H_12_O_3_	Resveratrol is derived from grapes	Rat glioma cell line C6. These cells are classified as part of the astrocyte lineage. The cells were treated with LPS at a concentration of 1 μg/mL. The treatments included exposure to LPS alone for 6 and 24 h, and combinations of LPS with resveratrol (25 μM) for 24 h.	Resveratrol: Decreases TAU hyperphosphorylation induced by LPS. Increases APP expression. Has a potential therapeutic role in reducing neuroinflammation and modifying the expression of proteins associated with AD.	[194]
Trans ε-viniferin Resveratrol.	Trans ε-viniferin—C_28_H_22_O_6_ Resveratrol—C_14_H_12_O_3_	Vine shoots (*Vitis vinifera*)	Murine primary co-cultures of neurons and astrocytes were prepared from the hippocampus and cortex of embryonic day 18 (E18) mice. The cultures were first pre-treated with either 1 μM of trans ε-viniferin or resveratrol, or with 0.001% DMSO as a vehicle control, in a medium with serum for 24 h. Following this pre-treatment, the cultures were then treated for 48 h in a serum-free medium with 20 μM A*β*42, which had been previously aggregated by incubation for 72 h at 37 °C, and 200 pg/mL IL-1*β* to mimic the inflammatory context of AD. The selection of the 1 μM concentration for the polyphenols was based on preliminary cytotoxicity assays that showed higher concentrations of trans *ε*-viniferin were cytotoxic, whereas 1 μM was not.	Trans ε-viniferin: Induces the disaggregation of A*β* peptide and rescues inflammation. These effects were higher than those of resveratrol, making trans ε-viniferin a promising multi-target therapeutic candidate for AD.	[195]
**Tannins**					
Tannic acid (TA) Epigallocatechin gallate (EGCG)	TA: C_76_H_52_O_46_ EGCG: C_22_H1_8_O_11_	TA: Found in various plant species, including oak, sumac, and witch hazel. Epigallocatechin gallate (EGCG): Major component of green tea leaves (*Camellia sinensis*)	SH-SY5Y neuronal cells Cells were treated with 20 μM concentrations of TA and EGCG	TA: Acts as a dual-acting therapeutic agent against AD and ferroptosis. Modulates A*β*42 and TAU aggregation. Chelates metal ions, reducing oxidative stress. Rescues mitochondrial function. Activates and enhances GPX4 levels, inhibiting ferroptosis. Activates Nrf2, regulating ferroptosis in neuronal cells. Inhibits lipid peroxidation and protein oxidation, providing neuroprotective effects. EGCG: Inhibits A*β*42 aggregation. Shows antioxidant properties, reducing oxidative stress. Does not directly activate GPX4 but inhibits RSL3-induced GPX4 inhibition.	[196]

AD: Alzheimer’s disease; A*β*: Amyloid-beta; BDNF: brain-derived neurotrophic factor; EC: epicatechin; CE: catechin; DHA: docosahexaenoic acid; EGCG: epigallocatechin-3-Gallate; APP: amyloid precursor protein; p-TAU: phosphorylated tau; ∆Ψm: mitochondrial membrane potential; DMSO: dimethyl sulfoxide; IL-1*β*: interleukin-1 beta; TA: tannic acid; GPX4: glutathione peroxidase 4; Nrf2: nuclear factor erythroid 2-related factor 2; LPS: lipopolysaccharide; PKC: protein kinase C; PUMA: p53-upregulated modulator of apoptosis; CASPASE-3: cysteine–aspartic proteases-3; WT: wild-type; PSEN1: presenilin 1; APOE: apolipoprotein E; TR: targeted replacement; KO: knock-out; SK-N-SH: human neuroblastoma cell line; HT-22: mouse hippocampal cell line; BE(2)-M17: human neuroblastoma cell line; PC12: rat pheochromocytoma cell line; SH-SY5Y: human neuroblastoma cell line; iPSC: induced pluripotent stem cell; NO: nitric oxide; SNAP: S-nitroso-N-acetylpenicillamine; TNF*α*: tumor necrosis factor alpha; DJ-1: protein deglycase DJ-1; NMDAR: N-methyl-D-aspartate receptor; PP2A: protein phosphatase 2A; GSK3: glycogen synthase kinase 3; MAF14001: anthocyanin/anthocyanidin formulation.

**Table 2:** In vivo studies on polyphenols: mechanisms of action in Alzheimer’s disease prevention.

Polyphenol	Chemical Formula	Plant Origin	Experimental Model	Health Effects	Reference
**Flavonoids**					
Catechin Epicatechin Proanthocyanidins		*Vitis vinifera* (Grape seed polyphenol extract—GSPE)	Male Sprague–Dawley rats. Rates aged 12 weeks and weighing between 250–260 g. GSPE was administered at doses of 25 or 250 mg/kg BW. The mode of administration was oral gavage, carried out once daily over a period of 11 days.	GSPE-derived phenolic acids, specifically 3-hydroxybenzoic acid (3-HBA) and 3-(3′-hydroxyphenyl) propionic acid (3-HPP): Interfere with the assembly of A*β* peptides into neurotoxic aggregates. This suggests potential therapeutic effects in modulating AD pathogenesis by preventing the formation of A*β* oligomers and fibrils.	[197]
Baicalin Icariin Dihydromyricetin (DHM) Hesperidin	Baicalin: C_21_H_18_O_11_ Icariin: C_33_H_40_O_15_ Dihydromyricetin (DHM): C_15_H_12_O_8_ Hesperidin: C_28_H_34_O_15_	Baicalin: *Scutellaria baicalensis* Icariin: Epimedium species Dihydromyricetin (DHM): *Hovenia dulcis* Hesperidin: *Citrus sinensis* and *Citrus reticulata*	Heterozygous male transgenic APP/PS1-(21) mice, bred with female wild-type C57BL/6J mice. Each polyphenol (baicalin, icariin, DHM, hesperidin) was administered at a dose of 100 mg/kg BW. The polyphenols were administered orally via gavage, dissolved in 1% carboxymethylcellulose (CMC). The treatment period was ten days, with daily administration of the polyphenols.	Hesperidin and icariin: Restore behavioral deficits. Reduce A*β* deposits in both the cortex and hippocampus of the transgenic mice. Baicalin and DHM: No substantial effects on the above parameters.	[198]
Gallic acid Catechin Epicatechin Proanthocyanidins	Gallic acid: C_7_H_6_O_5_ Catechin: C_15_H_14_O_6_ Epicatechin: C_15_H_14_O_6_ Proanthocyanidins: C_31_H_28_O	*Vitis vinifera* L. (grape seed extract (GSE) is rich in polyphenols)	APPSwe/PS1dE9 transgenic mice. Mice were administered daily polyphenols with a total polyphenolic content of 592.5 mg/g DW, including gallic acid (49 mg/g DW), catechin (41 mg/g DW), epicatechin (66 mg/g DW), and proanthocyanidins (436.6 mg catechin Eq./g DW). The daily consumption of polyphenols was approximately 1.2–1.7 mg per gram of BW, which is equivalent to about 5.9 g per day for a 60 kg human.	GSE: Reduces the A*β* burden in the brain and blood. Prevents A*β* deposition. Attenuates microgliosis and inflammation. Reduces brain A*β* levels by 33%, serum A*β* levels by 44%, amyloid plaques by 49%, and microgliosis by 70%.	[199]
**Stilbenes**					
Resveratrol	C_14_H_12_O_3_	Grapes, red wine, and mulberry (*Morus atropurpurea* L.)	Senescence-accelerated mouse prone 8 (SAMP8) and senescence-accelerated mouse resistant 1 (SAMR1). The diet was supplemented with trans-resveratrol at a concentration of 1 g/kg and administered ad libitum to mice, starting at 2 months of age and maintained on this regimen until 9 months of age.	Resveratrol: Increases lifespan and reduces neurodegenerative markers in SAMP8 mice. Reduces levels of A*β* peptides. Decreases tau hyperphosphorylation. Enhances cognitive function and memory in animal models. Activates SIRT1 and AMPK pathways, promoting non-amyloidogenic processing of APP. Reduces oxidative stress.	[200]
Resveratrol	C_14_H_12_O_3_		Double-transgenic A*β*PP/PS1 mice, which express a chimeric mouse/human A*β* protein precursor (Mo/Hu A*β*PP695swe) and a mutant human presenilin 1 (PS1-dE9). Mice were fed a chow diet containing 1% resveratrol, which translated to a daily resveratrol consumption of approximately 4 mg/kg/day, over a period of 10 months starting from 2 months of age.	Resveratrol: Prevents memory loss as measured by the object recognition test. Reduces the A*β* burden. Increases mitochondrial complex IV protein levels. These effects were mediated by increased activation of SIRT1 and AMPK pathways. Promotes changes in inflammatory processes (increased IL1*β* and TNF gene expression).	[201]
Resveratrol	C_14_H_12_O_3_		Male wild-type (WT) and AD transgenic 5xFAD mice. Mice were subjected to a high-fat diet (HFD) and resveratrol supplementation for 16 weeks, starting from 2 months of age to 6 months. The HFD constituted 60% of calories from fat, while the resveratrol-supplemented diet included 1 g/kg of trans-resveratrol (0.1% *w*/*w*). This concentration was chosen based on previous studies demonstrating its neuroprotective effects. The mice received approximately 120 mg/kg BW of trans-resveratrol per day.	Resveratrol: Neuroprotective effects against HFD-induced amyloid pathology, reducing amyloid burden and tau pathology in the cerebral cortex. Improves memory deficits and enhanced brain resilience against neurodegeneration through proteostasis enhancement and tau deacetylation mediated by SIRT1.	[202]
**Mix of polyphenols**					
Grape seed polyphenol extract (GSPE)		*Vitis vinifera* L. (Grape seeds)	TMHT mouse model of AD. Mice were treated with 200 mg/kg/day of GSPE, starting at 4 months of age and continued for 2 months.	GSPE: Interferes with the assembly of tau peptides into neurotoxic aggregates Reduces the development of AD-type tau neuropathology. Decreases tau phosphorylation at specific sites, thereby preventing the formation of NFTs and reducing ERK 1/2 activity. Led to a significant reduction in the accumulation of insoluble tau and hyperphosphorylation of tau in the brains of TMHT mice.	[203]
Flavonoids Phenolic acids Anthocyanins		Muscadine wine was generated from *Vitis rotundifolia* (cv. Noble grapes). Cabernet Sauvignon wine was generated from *Vitis vinifera* L. grapes.	Tg2576 AD mouse model mice. Mice consumed approximately 4 mL of wine-adulterated water per day over a period of 10 months, with the concentration of polyphenols in the muscadine wine measured as 1.731 mg/L (as gallic acid).	Muscadine wine polyphenols: Interfere with the aggregation of A*β* peptides into high-molecular-weight oligomeric species, which are implicated in cognitive dysfunction in AD. Cabernet Sauvignon wine polyphenols: Promote non-amyloidogenic *α*-secretase activity, reducing A*β* neuropathology.	[204]
Anthocyanins Flavonoids Organic acids (malic acid, oxalic acid, and tartaric acid)		*Vitis vinifera* L. (red grape),	Wistar rats (12–15 weeks old, weighing 250–300 g) AD was induced by orally administering AlCl3 at a dose of 17 mg/kg BW daily for four consecutive weeks. After the induction period, the rats were divided into five groups: normal healthy controls, normal rats receiving *Vitis vinifera* leaf polyphenol (VLP) extract, AD-induced positive controls, AD-induced rats treated with VLP extract, and AD-induced rats treated with rivastigmine (RIVA). The VLP extract was administered orally at a dose of 100 mg/kg BW daily for 21 days, whereas RIVA was administered at a dose of 0.3 mg/kg BW daily for the same duration.	VLP: Neurorestorative activity. Antiapoptotic activity. Anti-inflammatory activity. Anticholinesterase activity. Antioxidative activity. Anti-amnesic activity. Improves behavioral outcomes in T-maze tests. Reduces brain damage as evidenced by histopathological investigations.	[205]
Anthocyanins Hydroxycinnamic acid derivatives (e.g., caffeic acid) Hydrolyzable tannins (e.g., ellagic acid, quercetin-3-O-glucoside, punicalin) Hydroxybenzoic acids (e.g., gallic acid, protocatechuic acid) Hydroxycyclohexane carboxylic acids (e.g., quinic acid) Hydroxyphenyls (e.g., kaempferol, catechin)		*Punica granatum*	APPsw/Tg2576 transgenic mice were used alongside wild-type control mice. The experimental groups consisted of wild-type controls on a regular diet, transgenic controls on a regular diet, and transgenic mice fed with a 4% pomegranate-enriched diet. The pomegranate supplementation was administered for 15 months	Pomegranate: Attenuates oxidative damage, as evidenced by decreased lipid peroxidation and protein carbonyl levels. Restores the activities of antioxidant enzymes (superoxide dismutase, catalase, glutathione peroxidase, glutathione, and glutathione S transferase) in the brain. This suggests that the therapeutic potential of pomegranate in the treatment of AD might be associated with its ability to counteract oxidative stress.	[206]
Cranberry extract (CBE)		*Vaccinium macrocarpon*	*Caenorhabditis elegans* (C. elegans) transgenic strain CL4176. The polyphenol concentration used was 2 mg/mL of CBE. The administration of CBE was performed through supplementation in the nematode growth medium (NGM) plates. For the preventive treatment, CBE supplementation began from egg hatching and continued until the L3 larval stage, before the induction of A*β* expression via heat-shock. For the therapeutic treatment, CBE was administered starting from the L3 stage after A*β* expression induction. The incubation/administration time varied accordingly: in the preventive treatment, CBE exposure was until the L3 stage prior to A*β* induction, whereas in the therapeutic treatment, it was from L3 stage until paralysis occurred. Both protocols aimed to assess the efficacy of CBE in alleviating A*β* toxicity and its potential preventive versus therapeutic effects in the *C. elegans* AD model.	CBE: Delays the body paralysis triggered by A*β* toxicity. Reduces A*β* expression at both RNA and protein levels. Improves memory health.	[207]
Dark chocolate (DC) containing 4% total polyphenol content	One of the primary polyphenols in dark chocolate is epicatechin: C_15_H_14_O_6_	*Theobroma cacao*	Sprague–Dawley rat pups. Rats were divided into four groups: control rats (Ctrl), control rats treated with dark chocolate (Ctrl Choc), non-transgenic AD (NTAD) model rats induced by monosodium glutamate (MSG), and NTAD rats treated with dark chocolate (MSG Choc). The dark chocolate (DC) used contained 70% cocoa solids and 4% total polyphenol content, administered orally at a dosage of 500 mg/kg BW per day. The polyphenol content in the dark chocolate used in the study was determined to be 20 mg of total polyphenol content (TPC) in 500 mg of dark chocolate	Dark chocolate: Reduces hyperglycemia in the treated rats. Inhibits the cholinesterase activity in the hippocampal tissue homogenates. Enhances cognitive performance in the spatial memory-related Barnes maze task. Increases cell volume in the CA3 region of the hippocampus. Improves cognitive function and cholinergic activity in the hippocampus while correcting metabolic disturbances in aged NTAD rats.	[208]
*Harrisonia abyssinica*	Quercetin (C_15_H_10_O) Kaempferol (C_15_H_10_O_6_) Apigenin (C_15_H_10_O_5_)	*Harrisonia abyssinica*	Male Wistar albino rats. The rats were divided into five groups: a control group, an AlCl_:_treated group, a rivastigmine-treated group, and two groups treated with different doses of *Harrisonia abyssinica* extract. AlCl_3_ was administered at a dose of 100 mg/kg BW per day orally. Rivastigmine was administered at a dose of 0.3 mg/kg BW intraperitoneally. *Harrisonia abyssinica* extract was administered at two doses: 100 mg/kg BW and 200 mg/kg BW per day by oral gavage. The treatments were administered for a period of 3 weeks.	*Harrisonia abyssinica:* Improves memory and learning performance as assessed by the passive avoidance test. Reduces hippocampal levels of AChE and extracellular regulated kinase, both of which were elevated by AlCl_3_. Restores normal levels of neurotransmitters (noradrenaline, dopamine, serotonin) in the brain. Decreases oxidative stress markers, pro-inflammatory cytokines, and apoptotic markers. Prevents A*β* plaque deposition and restoration of normal histological appearance of the hippocampus.	[209]
Anthocyanin	Ccyanidin-3-glucosylrutinoside (C_27_H_31_O_15_), Cyanidin-3-rutinoside (C_27_H_31_O_15_), and Cyanidin-3-glucoside (C_21_H_21_O_11_)	Montmorency cherries	5xFAD transgenic mouse. The study administered a proprietary product, Total Body Rhythm (TBR), composed of tart cherry extract rich in anthocyanins (426.7 μg/mg) and omega fatty acids, to both 6-month-old and 12-month-old mice. The TBR treatment, dosed at 60 mg/kg, was delivered via oral gavage every other day for two months, with a 0.5% methyl-cellulose PBS solution as the vehicle	TBR: Reduces memory deficits in the MWM and NOR tests. Decreases anxiety levels in the OF task primarily in 6-month-old male mice. Protects against neuron loss, reduces activation of astrocytes and microglia primarily in 6-month-old mice, and attenuates A*β* deposition.	[210]
Cyanidin 3,5-O-diglucoside Cyanidin 3-O-glucoside Cyanidin 3-O-rutinoside (+)-Catechin (−)-Epicatechin Procyanidin dimer B1, B3, B4, and B5 Procyanidin trimer C1, C2, EEC, and T2 Kaempferol 3,7-O-diglucoside Kaempferol 3-O-(6″-acetyl-galactoside) 7-O-rhamnoside Kaempferol 3-O-galactoside Kaempferol 3-O-galactoside 7-O-rhamnoside	Cyanidin 3,5-O-diglucoside: C_27_H_31_O_16_ Cyanidin 3-O-glucoside: C_21_H_21_O_11_ Cyanidin 3-O-rutinoside: C_27_H_31_O_15_ (+)-Catechin: C_15_H_14_O_6_ (−)-Epicatechin: C_15_H_14_O_6_ Procyanidin dimer B1, B3, B4, and B5: C_30_H_26_O_12_ Procyanidin trimer C1, C2, EEC, and T2: C_45_H_38_O_18_ Kaempferol 3,7-O-diglucoside: C_27_H_30_O_16_ Kaempferol 3-O-(6″-acetyl-galactoside) 7-O-rhamnoside: C_29_H_32_O_16_ Kaempferol 3-O-galactoside: C_21_H_20_O_11_ Kaempferol 3-O-galactoside 7-O-rhamnoside: C_27_H_30_O_15_	*Rosa* x *hybrida*	*C. elegans* that express human A*β* peptide, a key protein involved in AD. Concentration used: 100 μg/mL. Worms were exposed to the extract in their growth medium.	*Rosa* x *hybrida*: Reduces paralysis rate significantly. Significant improvements in mobility were observed at 36, 38, 40, and 42 h post-induction. Increases the chemotactic index, which measures the worms’ ability to move towards a chemical stimulus. No toxicity was observed, as indicated by normal metabolism, reproduction, and lifespan parameters even after exposure to the *Rosa* x *hybrida* extract at a concentration of 100 μg/mL.	[211]
Cocoa polyphenols	Catechin: C_15_H_14_O_6_ Epicatechin: C_15_H_14_O_6_ Procyanidin B2: C_30_H_26_O_12_ Theobromine: C_7_H_8_N_4_O_2_	*Theobroma cacao*	*Caenorhabditis elegans*, the specific transgenic strain employed expressed A*β* in neurons, demonstrating middle-age onset behavioral dysfunction similar to human AD. For the polyphenol treatment, a concentration of 5 mg/mL cocoa powder, which contains 27.01 mg GAE/g of total phenolics and 10.13 mg CE/g of total flavonoids, was used. The administration method involved suspending the cocoa powder in M9 buffer with concentrated Escherichia coli OP50, which was then added to NGM plates. The worms were exposed to the cocoa treatment starting from the L1 larval stage and continued daily transfers onto fresh plates to avoid progeny production until they stopped laying eggs around day 9. Samples were collected at three different time points: days 4, 8, and 12, to represent young, middle, and old age, respectively.	Cocoa: Reduces A*β*-induced cognitive deficits. Reduces elevated levels of proline and asparagine in young transgenic worms, which are associated with cognitive deficits. Normalizes hypoxanthine levels in middle-aged transgenic worms, which is linked to memory deficits. Alters urea cycle metabolites, reducing ornithine levels in transgenic worms expressing A*β*, indicating potential modulation of AD pathology.	[212]
Ellagic acid Pelargonidin-3-glucoside Chrysanthemin Kaempferol-3-O-D-glucoside Pelargonidin-3-rutinoside	Ellagic acid: C_14_H_6_O_8_ Pelargonidin-3-glucoside: C_21_H_21_ClO_10_ Chrysanthemin: C_21_H_21_O_11_ Kaempferol-3-O-D-glucoside:C_21_H_20_O_11_ Pelargonidin-3-rutinoside: C_27_H_31_O_14_	Strawberry variety Fragaria × ananassa cv. Romina	Caenorhabditis elegans. The specific strains utilized included N2 Bristol (wild-type), CL4176 (expressing human amyloid *β*1–42 peptide), and others. Concentrations of 100, 500, and 1000 µg/mL of strawberry methanolic extract were used. Worms were exposed to these concentrations for various durations, depending on the specific assay, ranging from 15 min to several days.	Strawberry: Reduces A*β*-protein induced paralysis. Decreases A*β* aggregation. Prevents oxidative stress. The effects were mediated through the DAF-16/FOXO and SKN-1/NRF2 signaling pathways.	[213]
*Desmodium elegans*: Gallic acid Coumaric acid Chlorogenic acid Caffeic acid p-Coumaroylhexose 3-O-Caffeoylquinic acid 4-O-Caffeoylquinic acid (+)-Catechin Quercetin-3-rutinoside Quercetin-3-O-glucuronide Kaempferol-7-O-glucuronide Isorhamnetin-7-O-glucuronide Isorhamnetin-3-O-rutinoside	Gallic acid: C_7_H_6_O_5_ Coumaric acid: C_9_H_8_O_3_ Chlorogenic acid: C_16_H_18_O_9_ Caffeic acid: C_9_H_8_O_4_ p-Coumaroylhexose: C_15_H_18_O_8_ 3-O-Caffeoylquinic acid: C_16_H_18_O_9_ 4-O-Caffeoylquinic acid: C_16_H_18_O_9_ (+)-Catechin: C_15_H_14_O_6_ Quercetin-3-rutinoside: C_27_H_30_O_16_ Quercetin-3-O-glucuronide: C_21_H_20_O_13_ Kaempferol-7-O-glucuronide: C_21_H_18_O_13_ Isorhamnetin-7-O-glucuronide: C_22_H_20_O_13_ Isorhamnetin-3-O-rutinoside: C_28_H_32_O_16_	*Desmodium elegans*, which was collected from Ganajeer, Malamjaba Swat, Khyber Pakhtunkhwa, Pakistan	Mice. The mice were administered the test sample at a dose of 1 mg/kg per day (i.p.).	*Desmodium elegans* extracts: Improves cognitive performance, showing anxiolytic effects and enhancement in spatial memory. Improves the escape latency in the shallow water maze test, indicating enhanced memory and learning abilities in the treated mice compared to the disease control group.	[214]
Gallic acid Ferulic acid *p*-Coumaric acid	Gallic acid: C_7_H_6_O_5_ Ferulic acid: C_10_H_10_O_4_ *p*-Coumaric acid: C_9_H_8_O_3_	*Solanum lycopersicum* (tomato), *Cenostigma pluviosum*, and *Peltophorum dubium* represented 76.66% of the pollen types sampled	*Drosophila melanogaster* AD model (AD-like) Drosophila were treated with different concentrations of the methanolic pollen extract (0.1 mg/mL, 0.04 mg/mL, 0.02 mg/mL, and 0.004 mg/mL) over a period of up to 21 days. The administration was carried out using an enriched puree medium refreshed every two days.	Pollen: Enhances climbing ability of Drosophila melanogaster AD-like model flies. Reduces neurodegeneration index in histopathological analysis. Improves survival rate. Exhibits significant antioxidant response. Reduces damage in brain tissue.	[215]
**Phenolic acids**					
Chlorogenic acid (CGA)	C_16_H_18_O_9_	Tea, coffee, roasted green beans, berries, cocoa, citrus fruits, apples, and many vegetables	Male albino Swiss mice weighing 25–35 g. The mice received ICV-STZ injections at a concentration of 3 mg/kg on days 1 and 3 to induce SAD. Following the second STZ injection, CGA was administered at a concentration of 5 mg/kg orally, starting 2 h after the second STZ administration and continued daily for 26 days. The administration mode for CGA was oral gavage.	CGA: Alleviates memory deficits induced by ICV-STZ. Protects against an increase in nitrite/nitrate and TBARS levels in the brain. Preserves the number of viable cells in the prefrontal cortex and hippocampus. Prevents the depletion of BDNF in the prefrontal cortex and hippocampus. Mitigates astrogliosis and microgliosis in the prefrontal cortex and hippocampus. Exhibits neuroprotective effects, suggesting its potential as a therapeutic agent in the treatment of SAD.	[216]
**Curcuminoid**					
Curcumin (Cur) and Nanocurcumin (NC)	C_21_H_20_O_6_	*Curcuma longa*	B6SJL-Tg (5xFAD) mice. Cur and NC were dissolved in methanol and diluted with PBS to a final concentration of 50 mg/kg BW. These solutions were injected intraperitoneally once daily for either 2 or 5 days to one-year-old B6SJL-Tg (5xFAD) mice. The concentrations tested ranged from 1 mM to 1 nM to determine the minimum effective dose for labeling A*β* plaques.	Curc: Binds to A*β* plaques. Inhibits misfolded A*β* aggregation Reduces oxidative damage. Decreases A*β* plaques in the brain. NC: Shows enhanced permeability into brain tissue and binds to A*β* plaques more effectively than dietary Cu. Both Cu and NC can label A*β* plaques in postmortem and in vivo brain tissue, suggesting potential for monitoring A*β* plaque load after anti-amyloid therapy.	[217]
**Polyprenol or isoprenoid alcohol**					
Ropren^®^,	H-(C5H8)n-OH	*Picea abies* (L.) Karst	Male Wistar rats, aged 3–4 months and weighing between 180–200 g. The polyphenol being focusing on was Ropren^®^, a substance containing polyprenols extracted from the green verdure of Picea abies. Ropren^®^ was administered orally at a dosage of 8.6 mg/kg BW daily for 28 days. Additionally, testosterone propionate (TP) was administered subcutaneously at a dose of 0.5 mg/kg BW daily for the same period. The experimental procedure began with the induction of an AD model through the intracerebroventricular injection of A peptide (25–35) at a concentration of 3 μg/μL, followed by a 14-day recovery period. Subsequently, the rats underwent gonadectomy and another 14-day recovery period before starting the daily administration of Ropren^®^, TP, or oil solvent.	Ropren^®^: Ameliorates cognitive impairment induced by A*β* (25–35) peptide injection. Shows memory-enhancing effects Increases locomotor activity, rearing and grooming events, and completely restores impaired cognitive performance. Restores testosterone levels in the GDX/A*β* rats.	[218]
**Lignan**					
Magnolol (MN)	C_22_H_18_O_11_	*Magnolia officinalis*	TgCRND8 transgenic mice. Mice were fed with MN at concentrations of 20 mg/kg and 40 mg/kg via oral gavage. Donepezil, a commonly used AD medication, was administered at 5 mg/kg as a positive control. The treatments were carried out daily for a period of four consecutive months. The polyphenol and donepezil were dissolved in 0.5% sodium carboxymethyl cellulose (CMC-Na) for MN and in normal saline for donepezil.	MN: Ameliorates cognitive deficits. Suppresses neuroinflammation and synaptic dysfunction. Inhibits A*β* deposition. Modulates PI3K/Akt/GSK-3*β* and NF-κB pathways. Improves cognitive function through increased expression of synaptic proteins and anti-inflammatory cytokines.	[219]
**Ellagitannins**					
Ellagic acid (EA)	C_14_H_6_O_8_	Berries, nuts, and other fruits	Swiss albino male mice aged 8–12 weeks and weighing 26–30 g. Mice were housed under standard conditions and provided with food and water ad libitum. The mice were randomly divided into three groups: Control (vehicle), STZ-sAD (AD model), and STZ-sAD treated with EA. The sporadic AD model was induced through ICV injection of STZ at a dose of 3 mg/kg BW. EA was administered orally at a dose of 75 mg/kg BW for 28 days.	EA: Reverses the upregulation of AD biomarkers caused by STZ. Improves recognition memory as evidenced by the NORT test. Modulates genes involved in synaptic plasticity, such as AMPAR, and its scaffolding proteins. Reduces oxidative stress and neuronal loss. Enhances antioxidant enzyme activity and reduce lipid peroxidation. Downregulates apoptotic markers.	[220]

A*β*: amyloid-beta; AD: Alzheimer’s disease; AMPK: AMP-activated protein kinase; APP: amyloid precursor protein; APP/PS1: amyloid precursor protein/presenilin 1 (transgenic mouse model); APPsw/Tg2576: Swedish mutant amyloid precursor protein (transgenic mouse model); BDNF: brain-derived neurotrophic factor; BW: body weight; CA3: cornu ammonis region 3 (a region of the hippocampus); CBE: cranberry extract; DW: dry weight; EA: ellagic acid; ERK: extracellular signal-regulated kinases; GDX: gonadectomy; GSPE: grape seed polyphenol extract; HBA: hydroxybenzoic acid; HPP: hydroxyphenyl propionic acid; MWM: Morris water maze; NC: nanocurcumin; NFTs: neurofibrillary tangles; NGM: nematode growth medium; NOR: novel object recognition; NTAD: non-transgenic Alzheimer disease; PBS: phosphate-buffered saline; PI3K/Akt/GSK-3*β*: phosphoinositide 3-kinase/protein kinase B/glycogen synthase kinase 3 beta; SAD: sporadic Alzheimer’s Disease; SAMP8: senescence-accelerated mouse prone 8; SAMR1: senescence-accelerated mouse resistant 1; SIRT1: sirtuin 1; STZ: streptozotocin; TBR: Total Body Rhythm; TPC: total polyphenol content; TP: testosterone propionate; VLP: *Vitis vinifera* leaf polyphenols.

**Table 3:** In vitro and in vivo studies on polyphenols: mechanisms of action in Alzheimer’s disease prevention.

Polyphenol	Chemical Formula	Plant Origin	Experimental Model	Health Effects	Reference
**Flavonoids**					
Epigallocatechin-3-gallate (EGCG)	C_22_H_18_O_11_	*Camellia sinensis*	SweAPP N2a cells transfected with the human APP gene. Cells were treated with various nanoparticle formulations and controls (25 µM: 3 µM) for 18 h. Male Sprague Dawley rats, pre-cannulated and weighing between 200–250 g. Rats were administered 100 mg EGCG/kg BW via oral gavage, with blood samples collected over an 8 h period.	EGCG: Promotes non-amyloidogenic processing of APP by upregulating *α*-secretase, preventing brain A*β* plaque formation.	[221]
Epigallocatechin-3-gallate (EGCG) Luteolin Apigenin Naringenin Diosmin Flavone	EGCG: C_22_H_18_O_11_ Luteolin: C_15_H_10_O_6_ Apigenin: C_15_H_10_O_5_ Naringenin: C_15_H_12_O_5_ Diosmin: C_28_H_32_O_15_ Flavone: C_15_H_10_O_2_	EGCG: *Camellia sinensis* Luteolin: Many fruits, vegetables, and medicinal herbs Apigenin: Parsley, celery, and chamomile Naringenin: Citrus fruits Diosmin: Citrus fruits Flavone: Various plants including parsley and celery	N2a cells were stably transfected with the human APP-695 gene harboring the “Swedish” mutation (APPsw) Cells were treated with 1 µM EGCG for 48 h. APP/PS-1 double-mutant transgenic mice. EGCG was administered to these mice at a concentration of 10 mg/mL in their drinking water, resulting in an approximate dose of 37.1 ± 1.6 mg/kg/day. The treatment was carried out over a period of 5.5 months	EGCG: Reduces A*β* levels and plaques. Provides cognitive benefits in AD transgenic mice. Restores mitochondrial respiratory rates, membrane potential, ROS production, and ATP levels in brain mitochondria. Luteolin: Decreases ROS production. Restores mitochondrial membrane potential and ATP levels. Apigenin, naringenin, diosmin, and flavone: Reduce oxidative stress. Restore mitochondrial function including respiratory rates, membrane potential, and ATP levels.	[222]
Epigallocatechin (EGC) Epicatechin-3-gallate (ECG)	EGC: C_15_H_14_O_7_ ECG: C_22_H_18_O_11_	*Camellia sinensis*	Neuro-2a cells. Cells were treated with A*β*40 and Cu^2+^/Zn^2+^-A*β*40 aggregates to induce neurotoxicity. EGC and ECG were used at a concentration of 20 μM, maintaining a ratio of [A*β*40]:[Cu^2+^/Zn^2+^]:[EGC/ECG] at 1:2:2 with an incubation time of 24 h. APP/PS1 transgenic mice. Micewere administered ECG intravenously at a dose of 100 mg/kg during a study period of 2 months.	EGC and ECG: Reduce the aggregation of A*β*40 induced by Cu^2+^ and Zn^2+^. Inhibit the formation of *β*-sheet-rich A*β*40 aggregates. Alleviate the Cu^2+^- and Zn^2+^-induced neurotoxicity on N2a cells by reducing ROS production. ECG cross the BBB and reduce A*β* plaques in the brains of APP/PS1 mice, protecting neurons from damage.	[223]
Trilobatin (TLB)	C_21_H_24_O_10_	*Lithocarpus polystachyus*	HT22 hippocampal cells. The cells were treated with various concentrations of TLB, specifically 1, 5, and 10 μM. The incubation time for these treatments was 24 h. C57BL/6J wild-type (WT) mice and 3xFAD transgenic AD mice. Animals were treated with TLB dissolved in saline and administered via gavage. The TLB concentrations used were 10 mg/kg and 20 mg/kg, administered once daily for 12 weeks.	TLB: Protects 3xFAD AD model mice against A*β* burden, neuroinflammation, tau hyperphosphorylation, synaptic degeneration, hippocampal neuronal loss, and memory impairment. Suppresses glial activation by inhibiting the TLR4-MYD88-NFκB pathway, leading to a reduction in inflammatory factors TNF-*α*, IL-1*β*, and IL-6. Ameliorates cognitive deficits. Reduces tau and A*β* pathology. Modulates spine plasticity. Protects against neuronal loss, and inhibited gliosis.	[224]
Trilobatin (TLB)	C_21_H_24_O_10_	*Lithocarpus polystachyus*	BV2 microglial cells BV2 cells were treated with A*β*25-35 to induce an AD model. TLB was administered at concentrations of 12.5, 25, and 50 μM. The treatment period for the experiments involving BV2 cells was not explicitly detailed but was sufficient to measure cell viability and cytotoxicity. Two animal models were used: APP/PS1 transgenic mice. Mice were administered TLB at doses of 4 and 8 mg/kg/day via intragastric (i.g.) administration for a period of 3 months. Rats subjected to intracerebroventricular (ICV) injection of A*β*25-35: These rats were subsequently administered TLB at doses of 2.5, 5, and 10 mg/kg/day via i.g. administration for 14 days.	TLB: Improves cognitive deficits in both animal models. Ameliorates neuroinflammation and oxidative stress by inhibiting the HMGB1/TLR4/NF-κB signaling pathway and activating the SIRT3/SOD2 pathway, which helps to restore redox homeostasis and suppress neuroinflammation.	[225]
**Curcuminoid**					
Curcumin	C_21_H_20_O_6_	*Curcuma longa*	Differentiated SK-N-SH human neuroblastoma cells. Cells were treated with varying doses of NanoCurc™ at concentrations of 250 nM, 500 nM, 1 μM, 2.5 μM, and 5 μM. These cells were co-treated with 100 μM H_2_O_2_ simultaneously for 24 h. Athymic mice Mice were administered NanoCurc™ intraperitoneally at a dose of 25 mg/kg twice daily for four weeks.	Curcumin: Reduces oxidative damage and amyloid pathology in AD models. Shows anti-inflammatory properties, inhibits pro-inflammatory transcription factors, and may disaggregate A*β* plaques, reducing neuroinflammation and protecting against AD progression. NanoCurc™: Protects neuronally differentiated human SK-N-SH cells from ROS (H_2_O_2_) mediates insults and rescues cells previously insulted with H_2_O_2_. In vivo, decreases levels of H_2_O_2_, caspase 3, and caspase 7 activities in the brain, and increases glutathione concentrations.	[226]
**Anthocyanins and Related Compounds**					
Delphinidin 3-galactoside (Del) and cyanidin 3-galactoside (Cya)	Del: C_21_H_21_O_12_ Cya: C_21_H_21_O_11_	*Vaccinium myrtillus*	Mouse neuroblastoma Neuro2a cells. The cells were exposed to A*β* samples incubated with or without VMA for either 24 or 48 h. Specifically, A*β*1–40 solutions were incubated at 37 °C for either 48 or 96 h, and A*β*1–42 solutions were incubated for either 24 or 48 h before being added to the cell cultures. The VMA concentrations used in the cell culture studies were typically prepared at a 2.5-fold molar ratio relative to the A*β* peptides. The incubation times varied depending on the specific assay but generally included intervals up to 96 h for A*β*1–40 and 48 h for A*β*1–42 Double-transgenic mice expressing human APP with the Swedish mutation (K670N/M671L) and human presenilin-2 proteins containing the N141I mutation. The DT mice were fed a diet supplemented with 1% VMA. The administration started at 60 days after birth and continued throughout the study period. Additionally, a lower concentration of 0.25% VMA was also tested in some groups of DT mice.	VMA Prevents cognitive degeneration in AD mice. However, this cognitive improvement was paradoxically associated with an increase in insoluble A*β* deposits in the brain, suggesting that VMA might promote the formation of non-toxic A*β* aggregates, diverting them from forming toxic species.	[227]
Myricetin, Quercetin, and Anthocyanin-rich extracts	Myricetin: C_15_H_10_O_8_ Quercetin: C_15_H_10_O_7_	The polyphenols were derived from blackcurrants and bilberries	Human SH-SY5Y neuroblastoma cells stably overexpressing the APP751 isoform. Myricetin: 2 and 20 μM Quercetin: 10 μM Anthocyanin-rich extracts: 8 and 31 /mLμg/mL. Incubation time: 24 h for normal growth conditions and assessments of viability and APP processing, 60 min for ROS measurement under menadione-induced stress conditions. Male APdE9 mice and age-matched wild-type littermates. Bilberry extract: 1.53 mg/g. Blackcurrant extract: 1.43 mg/g	Bilberry and blackcurrant anthocyanin-rich extracts: Decrease APP C-terminal fragments levels Alleviate spatial working memory deficits and hyperactivity in the APdE9 mouse model of AD. These findings suggest that dietary polyphenols from bilberries and blackcurrants might have potential benefits in modulating neurodegenerative processes associated with AD.	[228]
**Mix of polyphenols**					
Grape Seed Polyphenol Extract (GSPE) Resveratrol	Resveratrol: C_14_H_12_O_3_	GSPE: Derived from grape seeds. Resveratrol: Found in grapes and wine.	Paired helical filaments (PHFs) isolated from AD brains. PHFs were treated with 100 µM GSPE for varying incubation times, ranging from 5 s to 24 h, with a standard incubation time of 1 h generally used to ensure robust results. TMHT mouse model of tauopathy. GSPE at concentrations ranging from 1 µM to 100 µM, and resveratrol at a concentration of 100 µM, was administered to the mice at a daily dose of 200 mg/kg BW for the duration of the animal experiment, which lasted four weeks.	GSPE: Disrupts and disintegrates the ultrastructure of native PHFs in AD brain. Inhibits tau aggregation and promotes dissociation from already assembled filaments. Significantly inhibits tau-mediated neuropathology in the TMHT mouse model. Suggested as a potential therapeutic agent for tau-mediated neurodegenerative conditions, like AD. Resveratrol: Ineffective in altering the ultrastructure of PHFs as compared to GSPE. Not effective in inducing width expansion of filaments.	[229]
Punicalagin and ellagic acid, both components of pomegranate extract (POMx)	Punicalagin: C_48_H_28_O_30_ Ellagic Acid: C_14_H_6_O_8_	*Punica granatum*	HeLa/NFAT stable cell line. Polyphenol concentration: 0.1 mmol/L to 100 mmol/L for both punicalagin and ellagic acid. Incubation time: 30 min pre-treatment followed by 6 h of stimulation with TPA and ionomycin. Male C57BL/6 APPswe/PS1dE9 transgenic mice. POMx at 6.25 mL/L in drinking water. Oral administration via drinking water for 3 months.	POMx: Improves behavioral performance. Reduces microgliosis. Decreases NFAT activity and lowers TNF-*α* concentrations in the brains of the APP/PS1 mice. These effects suggest that POMx has anti-inflammatory properties that may attenuate the progression of AD by reducing neuroinflammation and improving cognitive functions.	[230]
3-O-quercetin glucopyranoside Rutin 3-O-Quercetin rhamnopyranoside Chlorogenic acid	3-O-Quercetin Glucopyranoside (Isoquercetin): C_21_H_20_O_12_ Rutin (Quercetin-3-rutinoside): C_27_H_30_O_16_ Ursolic Acid: C_30_H_48_O_3_ Oleanolic Acid: C_30_H_48_O_3_ 3-O-Quercetin Rhamnopyranoside (Quercitrin): C_21_H_20_O_11_ Chlorogenic Acid: C_16_H_18_O_9_ Scopoletin: C_10_H_8_O_4_	*Bouvardia ternifolia* (BtHA)	Human neuroblastoma cell line SH-SY5Y. Cells were treated with various concentrations of fibrillar A*β* (Ab1-42) peptide in the presence of BtHA or its specific fractions. The primary bioactive constituents of BtHA included 3-O-quercetin glucopyranoside (415 mg/g), rutin (229.9 mg/g), ursolic acid (54 mg/g), oleanolic acid (20.8 mg/g), 3-O-quercetin rhamnopyranoside (12.8 mg/g), chlorogenic acid (9.5 mg/g), and scopoletin (1.38 mg/g). Male ICR-strain mice were employed to assess the anti-inflammatory activity using the TPA-induced ear edema assay. The BtHA extract and its fractions were administered topically at a dose of 6.5 mg per ear. The extract and fractions were applied 15 min after TPA treatment.	The study found that Bouvardia ternifolia extract exhibited significant neuroprotection against A*β* peptide, with anti-inflammatory, antioxidant, and anti-acetylcholinesterase effects. These effects are attributed to its content of polyphenols, coumarins, and triterpenes, suggesting potential as a therapeutic agent in the treatment of AD	[231]
Proanthocyanidins Hecogenin Ferulic acid Catechin Gallic acid Epicatechin Epicatechin gallate	Ferulic acid: C_10_H_10_O_4_ Catechin: C_15_H_14_O_6_ Gallic acid: C_7_H_6_O_5_ Epicatechin: C_15_H_14_O_6_	*Fagopyrum dibotrys* (FDE)	SH-SY5Y cells. Cells were treated with FDE at concentrations of 2.5 mg/mL, 5 mg/mL, and 10 mg/mL. The cells were incubated with these concentrations for 24 h to assess the neuroprotective effects against A*β*-induced toxicity. APP/PS1 transgenic mice. Mice were administered a diet containing 0.65% FDE for a period of nine months. The amount of FDE consumed by the mice was approximately 0.103 mg/kg/day, ensuring consistent and long-term exposure to the extract.	FDE: Cleans A*β* deposits in the brain. Decreases A*β* burden in the plasma. Inhibits microhemorrhage. Reduces reactive microglia. Promotes A*β* fibrils disaggregation. Inhibits neurotoxicity induced by A*β* in SH-SY5Y cells.	[232]
Caffeic acid Trans-ferulic acid Isorhamnetin Irilin B	Caffeic Acid: C_9_H_8_O_4_ Trans-Ferulic Acid: C_10_H_10_O_4_ Acanthoside B: C_34_H_44_O_20_ Isorhamnetin: C_16_H_12_O_7_ Irilin B: (requires specific details for accurate formula)	*Salicornia europaea* L.	BV-2 microglial cells. The cells were pre-treated with SE-EE at concentrations of 20, 100, and 200 µg/mL for 1 h, followed by LPS stimulation (200 ng/mL) for either 6 or 24 h. Male C57BL/6N mice, aged 8–9 weeks and weighing 24–27 g, were used. The mice were divided into five groups, receiving either 0.9% saline, scopolamine (2 mg/kg), SE-EE (50 or 100 mg/kg), or tacrine (10 mg/kg). SE-EE and tacrine were administered orally for 7 days prior to scopolamine injection. The total phenolic content in SE-EE was measured as 51.29 mg gallic acid equivalent per gram of sample, and the flavonoid content was 19.87 mg rutin equivalent per gram of sample.	SE-EE: Dose-dependently attenuates LPS-induced inflammation in BV-2 cell Improves cognitive function in scopolamine-induced amnesic mice by suppressing oxidative stress markers, regulating inflammatory cytokines and associated proteins expression, ameliorating p-CREB/BDNF levels, and promoting neurogenesis and neuron proliferation.	[233]
Caffeic acid Quercetin derivatives (Quercetin-exoside-rhamnoside, Quercetin-dirhamnoside) Kaempferol derivatives (Kaempferol-3-O-glucoside-7-O-rhamnoside, Kaempferol-3,7-O-dirhamnoside) Isorhamnetin-hexoside-rhamnoside Sinapic acid Luteolin Di-O-sinapoyl-*β*-glucose isomers	Caffeic acid: C_9_H_8_O_4_ Quercetin-rhamnoside-hexoside: C_27_H_30_O_16_ Kaempferol-3-O-glucoside-7-O-rhamnoside: C_27_H_30_O_15_ Quercetin-dirhamnoside: C_27_H_30_O_15_ Isorhamnetin-hexoside-rhamnoside: C_28_H_32_O_16_ Kaempferol-3,7-di-O-rhamnoside: C_27_H_30_O_14_ Sinapic acid: C_11_H_12_O_5_ Di-O-sinapoyl-*β*-glucose isomers: C_28_H_32_O_14_ Luteolin: C_15_H_10_O_6_	*Arabidopsis thaliana*	BV2 murine microglial cells. Cells were treated with 25 µM of aggregated A*β*25-35 peptide in the presence or absence of a polyphenolic extract from *Arabidopsis thaliana*. The extract was administered at a concentration of 20 µL/mL, derived from seedlings grown for seven days and cold-pressed to preserve the polyphenolic content. The incubation times for the treatments were set at 2 and 24 h. *Drosophila melanogaster* flies expressing human A*β*1–42 were employed. The flies were fed a diet supplemented with 40 µL/mL of the Arabidopsis extract throughout their developmental period.	*Arabidopsis thaliana:* Has significant anti-inflammatory effects both in vitro and in vivo. In BV2 cells, the extract reduces the expression of pro-inflammatory cytokines (IL-6, IL-1*β*, TNF-*α*) and increases the expression of anti-inflammatory cytokines (IL-4, IL-10, IL-13). These effects were observed after 24 h of treatment, indicating a robust anti-inflammatory response. The extract also activated the Nrf2-antioxidant response element signaling pathway, leading to upregulation of heme oxygenase-1 (HO-1) mRNA and increased NAD(P)H oxidoreductase 1 (NQO1) activity, which are indicators of enhanced cellular antioxidant defenses. In the Drosophila model, the polyphenolic extract significantly improves the impaired climbing ability of the AD flies expressing human A*β*1–42, confirming its neuroprotective effects in vivo. This suggests that the extract could mitigate the neurotoxic effects of A*β*, potentially offering a protective strategy against neurodegenerative diseases, like AD. These results highlight the potential of *Arabidopsis thaliana* polyphenolic extract as a therapeutic agent with anti-inflammatory and neuroprotective properties, warranting further investigation in more complex organisms to fully understand its impact on neuroinflammation and neurodegeneration.	[234]
Feruloylquinic acid p-Coumaroylquinic acid Rutin Quercetin-3(6″malonyl)-neohesperioside B-type procyanidin dimers Chlorogenic acids (CGAs) Flavan-3-ol glycosides Procyanidins	Feruloylquinic acid: C_17_H_20_O_9_ p-Coumaroylquinic acid: C_16_H_18_O_8_ Rutin: C_27_H_30_O_16_ Quercetin-3(6″malonyl)-neohesperioside: C_29_H_34_O_17_ Procyanidins: (C_15_H_14_O_6_)_n_ Chlorogenic acid: C_16_H_18_O_9_	*Humulus lupulu*s L.	Human neuroblastoma SH-SY5Y cells. Cells were treated with different concentrations of hop extracts or specific polyphenolic fractions. The polyphenol concentration used was 0.25 mg/mL for the total extract and concentrations varied for different fractions (e.g., 0.03 mg/mL for fraction B). The administration mode was direct incubation with the cell culture medium. For the cytotoxicity assay, SH-SY5Y cells were incubated with A*β*1–42 and hop extracts for 24 h. *Caenorhabditis elegans* model (CL2006) expressing human A*β*3-42. The nematodes were treated with hop extracts at concentrations ranging from 10 to 250 /mLμg/mL. The administration mode was oral feeding, and the incubation/administration time was 120 h.	*Humulus lupulu*s: Prevents A*β*1–42 aggregation and cytotoxicity. Exhibits antioxidant properties. Enhances autophagy, promoting the clearance of misfolded and aggregated proteins in SH-SY5Y cells. In vivo efficacy in reducing A*β*-induced toxicity in C. elegans models.	[235]
Polyphenol-rich fungi		*Phellinus linteus*, *Ganoderma lucidum*, and *Inonotus obliquus*	PC12 cells. These cells were treated with Zn^2+^ to induce A*β* aggregation. The cells were pretreated with different concentrations of the aqueous extract of mixed medicinal mushroom mycelia (MMMM) at 10 µg/mL (MMMM-L) and 100 µg/mL (MMMM-H) for 16 h before being exposed to 50 µM Zn^2+^ for 3 h. 5xAD transgenic mice. These mice were orally administered 30 mg/kg/day of MMMM for 8 weeks. Behavioral tests, including the Y-maze test (Y-MT) and the passive avoidance test (PAT), were conducted to assess memory function.	MMMM: In PC12 cells, MMMM treatment reduces A*β* aggregation, oxidative stress, and apoptosis while enhancing cell viability and antioxidant enzyme activity. In 5xFAD mice, MMMM treatment ameliorates memory impairments, reduced A*β* plaque accumulation, and decreased neuroinflammation in the hippocampus.	[236]
**Hydroxycinnamic acids**					
Rosmarinic Acid (RA)	C_18_H_16_O_8_	Lamiaceae family, including rosemary and lemon balm.	BBB model using brain capillary endothelial cell monolayers. The experiments included controls, like caffeine and sodium fluorescein, to establish permeability benchmarks. For the permeability assay, the cells were incubated with RA, and permeability was measured over a period of 20 min at intervals. 5-month-old female Tg2576 mice (AD model) and 7–10-week-old female C57BL/6J mice. The Tg2576 mice were divided into control and RA-fed groups for a period of 10 months. The C57BL/6J mice were divided into a control group and a group fed with a diet containing 0.5% RA for 7 weeks. Tg2576 mice: RA was included in the diet at an unspecified concentration for 10 months. C57BL/6J mice: 0.5% RA was added to the diet and administered for 7 weeks. The administration was oral through dietary inclusion.	RA: Increases the concentration of monoamines (dopamine, norepinephrine, 3,4-dihydroxyphenylacetic acid, and levodopa) in the cerebral cortex of mice. Downregulates the expression of MAO B in the substantia nigra and ventral tegmental area, regions involved in dopamine synthesis. These changes were linked to a suppression of A*β* aggregation in the brains of the AD model mice, suggesting that RA has a potential therapeutic effect against AD by enhancing the dopamine-signaling pathway and inhibiting A*β* aggregation.	[237]
*p*-Coumaric acid	C_9_H_8_O_3_	Many fruits, vegetables, and cereals	PC12 neuronal cells. The concentration of pCA used was 50 µg/mL, and the cells were incubated for three days with A*β*42 (10 µM) at 37 °C. *Drosophila melanogaster* (fruit fly). Various concentrations of pCA (50 µM, 100 µM, 500 µM, and 1000 µM) were administered through feeding. The administration was performed using a capillary feeder assay, and the effects were observed over a period corresponding to the lifespan of the flies, with specific attention paid to survival rate and locomotive ability.	*p*-Coumaric acid: Reduces A*β*42 fibrillation and decreases A*β*42-induced cell mortality in PC12 neuronal cells. In the Drosophila AD model, *p*-Coumaric acid partially reverses the rough eye phenotype, significantly lengthened lifespan, and enhanced mobility in a sex-dependent manner.	[238]
**Tannins**					
Tannic Acid (TA)	C_76_H_52_O_46_	TA is a plant-derived hydrolyzable tannin polyphenol found in numerous herbaceous and woody plants.	SweAPP N2a cells. These cells were treated with varying concentrations of TA (1.563, 3.125, 6.25, 12.5, or 25 μM) for 12 h to evaluate the effects on A*β* production and APP metabolism. Transgenic PSAPP mouse model. These mice were orally administered TA at a concentration of 30 mg/kg/day via gavage for 6 months. The study assessed cognitive function and AD-like pathology in these mice, including behavioral impairments, brain amyloid deposits, and neuroinflammation.	TA: Prevents behavioral impairments, such as hyperactivity, decreased object recognition, and defective spatial reference memory. reduces brain parenchymal and cerebral vascular amyloid deposits, and mitigates neuroinflammation. Decreases the production of various A*β* species, including oligomers. These effects were linked to the inhibition of BACE1 expression and activity, and a shift towards non-amyloidogenic APP processing pathways, suggesting that TA might serve as a potential prophylactic treatment for AD.	[239]
**Xanthones**					
Alpha-mangostin	C_24_H_26_O_6_	*Garcinia mangostana*	BV-2 microglial cells, Chang liver cells, and bEnd.3 mouse brain endothelial cells. Cells were treated with *α*-mangostin (*α*-M) or its nanoparticle formulation (NP(*α*-M)). The concentrations used were 25, 50, and 100 ng/mL for BV-2 cells and 50, 500, and 1000 ng/mL for Chang liver cells. These cells were treated for 24 h, followed by incubation with 2 μg/mL A*β* 1-42 (A*β*1–42) in serum-free DMEM for an additional 24 h. Male SAMP8 mice, female APP/PS1 transgenic mice, and Kun-Ming mice. Animals were administered intravenous injections of either *α*-M solution or NP(*α*-M) at a dosage of 1 mg/kg/day over a period of four weeks.	*α*-mangostin: Upregulates LDLR expression. Increases cellular uptake and degradation of A*β*1–42. Enhances brain clearance of A*β*1–42. Reduces A*β* deposition. Attenuates neuroinflammatory responses. Ameliorates neurologic changes. Reverses behavioral deficits in AD model mice.	[240]
**Avenanthramides**					
Avenanthramide-C (Avn-C)	C_17_H_15_NO_4_	*Avena sativa*	Hippocampal slices prepared from wild-type (C57BL/6J) and AD transgenic mice (Tg2576 and 5xFAD). The slices were treated with different concentrations of Avn-C (specifically 50 µM) and exposed to 0.5 µM oligomeric A*β*42 to examine the effects on long-term potentiation (LTP). The slices were incubated in artificial cerebrospinal fluid (aCSF) perfused with a gas mixture of 95% O_2_ and 5% CO_2_ at room temperature. The treatment duration for Avn-C was 2 h, with a 0.5-h pretreatment period before the addition of A*β*42 Wild-type C57BL/6J mice, Tg2576, and 5xFAD transgenic mouse models of AD. Male mice aged 7–8 months for Tg2576 and 5–6 months for 5xFAD Avn-C was administered orally at a concentration of 6 mg/kg per day for a period of 2 weeks.	Avn-C: Improves memory and cognitive functions in the transgenic mouse models of AD. Reverses the impaired LTP in both ex vivo- and in vivo-treated AD mice hippocampus. Improves recognition and spatial memory. Reduces caspase-3 cleavage. Reverses neuroinflammation. Increases levels of phosphorylated glycogen synthase kinase-3*β* (pS9GSK-3*β*) and IL-10. These beneficial effects are mediated through the binding of Avn-C to *α*1A adrenergic receptors, stimulating AMPK.	[241]

A*β*: amyloid-beta; AD: Alzheimer’s disease; *α*-M: alpha-mangostin; AMPK: AMP-activated protein kinase; APP: amyloid precursor protein; ATP: adenosine triphosphate; Avn-C: avenanthramide-C; BBB: blood–brain barrier; BW: body weight; ECG: epicatechin-3-gallate; EGC: epigallocatechin; EGCG: epigallocatechin-3-gallate; FDE: Fagopyrum dibotrys extract; GSPE: grape seed polyphenol extract; HMGB1: high-mobility group box 1; HO-1: heme oxygenase-1; IL: interleukin; LTP: long-term potentiation; MAO B: Monoamine oxidase B; NanoCurc™: nanoparticle formulation of curcumin; NFκB: nuclear factor kappa-light-chain-enhancer of activated B cells; NQO1: NAD(P)H quinone dehydrogenase 1; Nrf2: nuclear factor erythroid 2-related factor 2; N2a: neuro-2a, a murine neuroblastoma cell line; PAT: passive avoidance test; PHFs: paired helical filaments; POMx: pomegranate extract; RA: rosmarinic acid; ROS: reactive oxygen species; SE-EE: Salicornia europaea ethanol extract; SIRT3: sirtuin 3; SK-N-SH: human neuroblastoma cell line; SweAPP: Swedish amyloid precursor protein; TA: tannic acid; TLB: trilobatin; TNF-*α*: tumor necrosis factor-alpha; TPA: 12-O-Tetradecanoylphorbol-13-acetate; VMA: Vaccinium myrtillus anthocyanins; WT: Wild-type; Y-MT: Y-maze test.

The main limitations of these in vivo studies of polyphenols in the context of AD include several key issues. One significant limitation is that the doses of polyphenols used in these studies are often much higher than those consumed by humans through diet or supplements, raising concerns regarding the feasibility and safety of such high doses for human use. The mode of administration used in these studies, such as oral gavage or injections, also differs from typical human consumption, potentially affecting the bioavailability and efficacy of polyphenols [242]. Another limitation is the short duration of several studies, which may not accurately reflect the long-term effects of polyphenol consumption on AD progression. Furthermore, the complexity of AD, which involves multiple pathways and factors, makes it challenging to solely attribute the observed benefits to polyphenol intake. Finally, there is a lack of standardized methods and consistent protocols across studies, making it difficult to compare the results and draw definitive conclusions. These limitations suggest that, while in vivo studies provide valuable insights, further research, including well-designed human clinical trials, is necessary to fully understand the potential of polyphenols in preventing and treating AD.

While in vitro studies establish the foundational mechanisms of polyphenol action, in vivo studies confirm their efficacy in more complex biological systems and highlight practical considerations for their use in treating AD. Combining insights from both research types strengthens the case of polyphenols as promising candidates for preventing and delaying AD.

### 4.3. Preclinical Discoveries: Polyphenol Activity in Alzheimer’s Models

Polyphenols, a diverse group of naturally occurring compounds, have gained significant attention for their potential neuroprotective effects, particularly in the context of neurodegenerative diseases, such as AD. The presence of aromatic rings and hydroxyl groups in their structures is associated with potent antioxidant properties, which are believed to contribute to their neuroprotective effects [243]. Specifically, the structural features of polyphenols allow them to scavenge ROS, chelate metal ions, and modulate signaling pathways that can mitigate oxidative stress and inflammation, which are key factors in AD pathogenesis [244,245].

According to the results of in vitro and in vivo studies, one primary mechanism by which polyphenols may prevent AD is, as expected, their antioxidant activity, which protects neurons from oxidative stress (Table 1, Table 2 and Table 3). Polyphenols neutralize free radicals, reducing oxidative damage and potentially slowing neurodegenerative processes [186,246]. Another important mechanism involves their anti-inflammatory effects. Chronic inflammation is a key aspect of AD pathology, and polyphenols modulate various inflammatory pathways, reducing the levels of pro-inflammatory cytokines and thereby mitigating neuroinflammation, which is believed to contribute to AD progression [184,185]. Polyphenols also influence the production and aggregation of A*β* plaques. Studies have suggested that polyphenols can inhibit plaque formation or promote their clearance from the brain, thus potentially reducing their detrimental effects [188,195]. They also promote neuroprotection by enhancing neuronal survival and supporting neurogenesis through the modulation of neurotrophic factors, such as brain-derived neurotrophic factor (BDNF), which plays a critical role in neuronal health and cognitive function [186]. Furthermore, polyphenols bind and chelate metal ions, such as iron and copper, which are involved in generating free radicals through Fenton reactions. By binding to these metals, polyphenols reduce the oxidative stress and neuronal damage associated with AD [187]. Some polyphenols also affect the activities of enzymes involved in AD pathology. They can inhibit *β*-secretase and γ-secretase, which are enzymes involved in A*β* production, and influence acetylcholinesterase activity, which is relevant for cognitive function [188,191].

From in vitro studies (Table 1 and Table 3) it seems that epigallocatechin-3-gallate (EGCG), a polyphenol derived from *Camellia sinensis* (green tea), is distinguished among polyphenols owing to its neuroprotective effects. Using human SH-SY5Y neuroblastoma cells and Chinese hamster ovary cells transfected with human APP695, it was shown that EGCG exhibits potent iron-chelating activity comparable to that of desferrioxamine, a chelating agent. Treatment with EGCG significantly reduced both APP and toxic A*β* peptide production, and promoted non-amyloidogenic APP processing by increasing the secretion of soluble APP-*α* and activation of PKC (Table 1) [187]. Another study involving cholinergic-like neurons (ChLNs) derived from umbilical cord mesenchymal stem cells exposed to EGCG demonstrated that it inhibits APP aggregation and blocks the phosphorylation of tau. EGCG also increases mitochondrial membrane potential (∆Ψm) and decreases oxidation of DJ-1 at Cys106-SH. Furthermore, EGCG inhibits the activation of transcription factors c-JUN and P53, as well as PUMA and CASPASE-3, in mutant ChLNs compared to wild-type cells. It also reverses Ca^2+^ influx dysregulation and inhibits the activation of the transcription factor NF-κB, thereby reducing the secretion of the pro-inflammatory cytokine IL-6 in wild-type astrocyte-like cells (Table 1) [188]. Additionally, using SweAPP N2a cells transfected with the human APP gene, it was demonstrated that EGCG promotes non-amyloidogenic processing of APP by upregulating *α*-secretase, thereby preventing brain A*β* plaque formation. This effect was significantly enhanced when EGCG was delivered via nano-lipidic particles, which improved its bioavailability and ability to induce *α*-secretase activity, highlighting its potential as a treatment for AD (Table 2) [221]. N2a cells transfected with the human APP-695 gene harboring the “Swedish” mutation were treated with EGCG. The results showed that EGCG reduced A*β* levels and plaques, provided cognitive benefits, and restored mitochondrial respiratory rates, membrane potential, ROS production, and ATP levels in the brain mitochondria. These findings suggest that EGCG can mitigate oxidative stress and improve mitochondrial function (Table 3) [222]. In vivo studies further support the neuroprotective properties of EGCG. For instance, APP/PS1 transgenic mice treated with EGCG at a concentration of 10 mg/mL in their drinking water for over 5.5 months showed significant reductions in A*β* levels and plaques. Additionally, EGCG administration improved cognitive function and restored mitochondrial health, as evidenced by normalized respiratory rates, membrane potential, and ATP levels in brain mitochondria (Table 3) [222]. A study involving male Sprague–Dawley rats demonstrated that EGCG administered at 100 mg/kg body weight via oral gavage promoted the non-amyloidogenic processing of APP by upregulating *α*-secretase, effectively preventing the formation of brain A*β* plaques (Table 3) [221]. In another in vivo study with APP/PS1 transgenic mice, EGCG administered intravenously at a dose of 100 mg/kg for two months crossed the BBB and reduced A*β* plaques in the brain. This treatment protects neurons from Cu^2+^-and Zn^2+^-induced neurotoxicity by reducing ROS production and inhibiting the formation of *β*-sheet-rich A*β*40 aggregates. These results illustrate EGCG’s ability of EGCG to mitigate metal-induced A*β* aggregation and associated neurotoxicity, providing a multifaceted approach to neuroprotection (Table 3) [223]. EGCG’s potential as a therapeutic agent for AD can be attributed to its unique chemical structure. This structure enables EGCG to effectively scavenge ROS, chelate metal ions, and modulate various signaling pathways [247,248]. EGCG’s multiple hydroxyl groups contribute to its potent antioxidant activity, which is crucial for mitigating oxidative stress [247,249]. Additionally, EGCG’s ability to cross the BBB allows it to exert its effects directly on the central nervous system [250]. Interestingly, while EGCG shares some common features with other polyphenols, such as the presence of phenolic rings, its specific arrangement of hydroxyl groups and galloyl moiety enhances its biological activity [249,251]. This structural configuration not only increases its antioxidant capacity but also influences its interaction with proteins and enzymes involved in neurodegenerative processes [247,251].

Catechin and epicatechin, both polyphenols commonly found in *Theobroma cacao* (cocoa), have been studied for their neuroprotective properties in vitro and in vivo. One study on mouse hippocampal HT-22 cells and primary neuronal cultures showed that a cocoa polyphenolic extract containing catechin and epicatechin exerts neuroprotective effects by activating the BDNF survival pathway. This activation countered the neurite dystrophy induced by A*β* plaques and oligomers, suggesting the potential of cocoa polyphenols as preventive agents against neurodegenerative diseases, such as AD (Table 1) [186]. Another study using mouse brain hippocampal slices demonstrated that cocoa extracts reduced A*β* oligomer formation, prevented synaptic deficits, and restored long-term potentiation reduced by oligomeric A*β*, highlighting their role in maintaining synaptic health and cognitive function (Table 1) [246]. In vivo studies have also supported the beneficial effects of catechin and epicatechin. For instance, a study using APP/PS1 transgenic mice, which model AD, showed that the daily consumption of polyphenols, including catechin and epicatechin, significantly reduced the A*β* burden in the brain and blood, prevented A*β* deposition, and attenuated microgliosis and inflammation. Polyphenol treatment resulted in a substantial reduction in brain and serum A*β* levels, amyloid plaques, and microgliosis, demonstrating their potential to ameliorate AD pathology and improve cognitive outcomes (Table 2) [199]. In vitro studies have shown that quercetin reduces apoptosis, increases the expression of protective genes, such as PP2A, GSK3, and NMDAR, and modulates pathways involved in AD (Table 1) [190]. In vivo studies corroborate these findings by demonstrating the efficacy of quercetin in reducing A*β* burden, preventing amyloid deposition, and attenuating inflammation and cognitive deficits in animal models of AD (Table 2 and Table 3) [206,209,214,228,231,234,235].

Resveratrol is a polyphenol with a significant potential to prevent and/or delay AD. One in vitro study involved HEK293 cells stably transfected with human APP695, in which resveratrol was found to decrease the levels of both secreted and intracellular A*β* peptides. This reduction was achieved by promoting intracellular degradation of A*β* via a mechanism involving the proteasome. Additionally, the anti-amyloidogenic activity of resveratrol was demonstrated to be proteasome-dependent, suggesting that it enhances the clearance of A*β* peptides, potentially mitigating the formation of amyloid plaques (Table 1) [193]. It has also been tested in various experimental models including senescence-accelerated mouse prone 8 (SAMP8) [200], double-transgenic A*β*PP/PS1 [201], and AD transgenic 5xFAD mice [202]. The results consistently show positive effects on cognitive function, neuroprotection, and a reduction in AD markers [200,201,202]. Resveratrol works through several mechanisms, such as by reducing the levels of A*β* peptides, decreasing tau hyperphosphorylation, and activating SIRT1 and AMPK pathways, which are associated with increased longevity, enhanced cognitive function, and reduced oxidative stress [200,201,202]. Resveratrol has also been shown to enhance cognitive function and memory in animal models, reduce oxidative stress, inflammation, and amyloid deposition, and increase lifespan while promoting the non-amyloidogenic processing of APP [200,201,202]. In addition to targeting AD markers, resveratrol improves mitochondrial function, enhances proteostasis, and promotes general brain health (Table 2) [200,201,202].

In summary, preclinical studies have indicated that polyphenols offer significant health benefits in AD. These compounds have been shown to reduce A*β* plaque formation, decrease oxidative stress, and improve cognitive function in animal models. Additionally, cell line studies have provided insights into the mechanisms by which polyphenols exert their neuroprotective effects, including anti-inflammatory actions and the enhancement of neuronal survival. Collectively, these findings suggest that polyphenols could be promising therapeutic agents in the fight against AD.

### 4.4. Preclinical Discoveries: Olive-Derived Polyphenol Activity in Alzheimer’s Models

The chemical structures of EGCG, resveratrol, and catechin and epicatechin previously discussed differ from olive oil polyphenols in ways that influence their effects on AD pathology. These structural differences impact their antioxidant activity, ability to inhibit A*β* aggregation, neuroprotective effects, and capacity to cross the BBB. Olive oil polyphenols, like HT and OC, generally have simpler structures compared to the others. Catechin and epicatechin are flavanols with similar structures, differing only in the spatial orientation of a hydroxyl group. EGCG, also a flavanol, has a more complex structure with an additional galloyl group. Resveratrol, a stilbene, has a distinct structure with two phenol rings connected by a double-bonded ethylene bridge. These structural variations lead to different effects on AD pathology.

**Figure 8:**
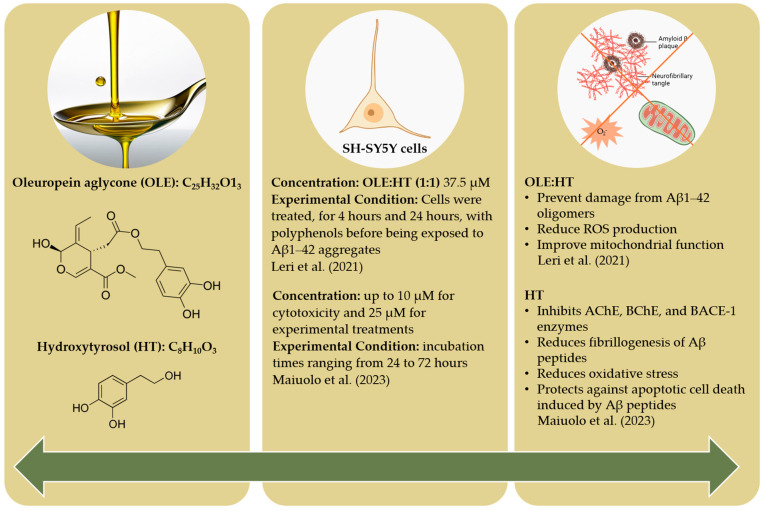
In vitro studies on olive-derived polyphenols: mechanisms of action in Alzheimer’s disease prevention. OLE: oleuropein aglycone; HT: hydroxytyrosol; SH-SY5Y: SH-SY5Y cells; ROS: reactive oxygen species; A*β*1–42: amyloid beta 1–42; AChE: acetylcholinesterase; BChE: butyrylcholinesterase; BACE-1: beta-site APP-cleaving enzyme 1. Leri et al. (2021) [252] https://www.mdpi.com/1422-0067/22/13/7225, accessed on 8 October 2024. Maiuolo et al. (2023) [253] https://www.mdpi.com/1422-0067/24/17/13461, accessed on 8 October 2024.

Oleuropein aglycone (OLE) and HT from EVOO synergistically activate autophagic flux in human neuroblastoma SH-SY5Y cells, protecting them from A*β*1–42 oligomer-induced damage, reducing ROS production, and improving mitochondrial function, highlighting their potential in treating neurodegeneration associated with AD (Figure 8) [252]. HT and its hybrid compounds have also shown promising results by inhibiting key enzymes implicated in AD pathology and reducing oxidative stress and fibrillogenesis in neuronal cells (Figure 8) [253]. OC from EVOO has been studied in human brain endothelial cells and transgenic mouse models. Additionally, a mixture of Greek and Saudi Arabian EVOO with distinct phenolic composition demonstrated the ability to reduce A*β* peptide aggregation and fibril mass, with lower concentrations of polyphenols needed in comparison to when Greek EVOO was used individually. Nevertheless, the extracts were less effective in reducing tau aggregation, as higher concentrations of bioactive compounds were required [254]. In vivo, OC significantly reduced the A*β* burden in TgSwDI mice and facilitated A*β* clearance across the BBB, suggesting its therapeutic potential by targeting multiple AD-related pathological mechanisms [81,255]. Additionally, OC improves cognitive function and reduces neuroinflammation in 5xFAD mice, demonstrating its efficacy in attenuating A*β* plaques and tau phosphorylation [256]. Another study on an EVOO-rich diet in a hTau mouse model showed significant improvements in working memory, spatial learning, and synaptic activity. Chronic administration of EVOO also reduces phosphorylated tau and tau oligomers, indicating better neuronal health and synaptic communication [257]. Similarly, dietary supplementation of olive oil, a rich source of OLE, could be effective in prevention and treatment of AD [258].OLE exhibits beneficial effects in TgCRND8 mice by improving memory function, enhancing autophagy, and reducing amyloid plaque deposition and size [259,260]. In a different experimental model using *Caenorhabditis elegans*, OLE effectively reduced A*β*-induced paralysis and plaque formation, further demonstrating its potential in combating AD-related neurotoxicity [261]. Studies in N2a neuroblastoma cells and TgCRND8 mice have reinforced OLE’s protective effects of OLE against DNA damage, neuroinflammation, and A*β* pathology by modulating key molecular pathways [260,262]. Finally, a study on polyphenols isolated from *Corallodiscus flabellata* revealed their ability to improve cognitive dysfunction and ameliorate neuronal damage and oxidative stress in both PC12 cells and Kunming mice, showing their potential for AD treatment by enhancing mitochondrial function and reducing A*β* levels [263].

### 4.5. Clinical Trials

As demonstrated, evidence suggests the preventive and therapeutic roles of polyphenols on AD. Olive oil and its by-products are common sources of these compounds that exhibit remarkable antioxidant and anti-inflammatory properties. Nevertheless, there is an emerging need to support information obtained through preclinical studies with clinical trials.

Polyphenols have been widely used in clinical trials, with their application dating back to at least 2010 [264]. In this review paper, we will focus on a select number of studies, particularly those that evaluate the effects of phenolic compounds from natural sources on AD and mild cognitive impairment (MCI), which frequently progresses to AD. The effects of the consumption of green tea on cognitive function and oxidative stress have been assessed in 30 patients with AD. The results demonstrate the capacity of polyphenols present in green tea, namely catechins, to overall increase endogenous antioxidant capacity, reducing the levels of 8-hydroxy-2′-deoxyguanosine, a marker of DNA oxidative damage. Additionally, the authors registered a slight improvement on cognitive tests, demonstrating the potential of green tea consumption to minimize the dysregulation of cognitive behavior that is commonly associated with AD [265,266]. In another non-blinded and non-placebo controlled study, green tea demonstrated the ability to improve cognitive dysfunction in elderly individuals due to its numerous bioactive compounds, such as catechins and theanine [267]. In a follow-up study, the same group of investigators conducted a randomized, placebo-controlled trial on the effects of green tea. Contrary to their initial findings, this trial found that consuming green tea for 12 months did not lead to a significant improvement in cognitive test performance. However, this natural dietary component did demonstrate the ability to regulate oxidative stress levels in the elderly participants [268]. Moreover, Baum et al. evaluated the effects of curcumin on AD. This polyphenol constitutes approximately 5% of turmeric and contributes to its spicy flavor. Several biochemical markers and cognitive tests were conducted. Although no side effects were detected, the trial did not yield positive outcomes, as no statistically significant effect of curcumin on the aggregation of A*β* peptide was registered. In fact, the placebo group did not present cognitive deterioration during the time in which the trial was conducted, which, as expected, conditioned the detection of improvement in the treatment group. Nevertheless, this polyphenol was able to increase vitamin E, contributing to antioxidant capacity [269]. The absence of positive effects of curcumin was further corroborated years later by Ringman et al., who conducted a subsequent trial to assess the effects of a curcumin complex on AD. The authors suggest the reduced bioavailability as a possible explanation for the disparities between results in vivo and in clinical trials [270]. In a distinct trial, Desideri et al. evaluated the effect of flavanols of cocoa on individuals with MCI and found that the treatment improved the timeframe needed to complete certain cognitive and verbal tests, an effect that the authors attribute to increased insulin sensitivity, suggesting a fundamental role of glucose metabolism in cognitive processes. Moreover, blood pressure was reduced upon treatment, and other metabolic markers showed improvements [271]. In the GENIAL trial, the consequences of daily administration of genistein on early-onset AD patients were analyzed and the results demonstrate that the phenolic compound increased performance in two neurocognitive tests and AD patients with treatment did not show an increase in A*β* deposition in contrast to the individuals without treatment [272]. In older individuals with MCI, supplementation with concord grape juice resulted in improvement in verbal learning, with no side effects. Additionally, a slight rise in fasting insulin levels was observed among those who drank grape juice [273]. Also in adults with MCI, supplementation with a polyphenol rich grape and blue berry extract (Memophenol ^TM^) for 6 months allowed for faster information processing and visuospatial learning and improvements in other cognitive measures [274]. Moreover, an antioxidant beverage rich in polyphenols was administered for 8 months to AD patients, and this treatment resulted in lower levels of plasma total homocysteine, which is commonly elevated in these patients due to oxidative stress [275]. Finally, although in vitro and in vivo evidence suggests otherwise, soy isoflavanoids at a dosage of 100 mg/day did not enhance cognitive function in older adults with AD. Nevertheless, the authors emphasize the need for further research into the metabolism of this polyphenol to support safer and more effective clinical trials [276].

There are several clinical trials using olive oil or EVOO. For instance, in an ongoing study, the authors aim to evaluate the effects of EVOO on gene expression and metabolic pathways related with AD in healthy subjects with a family history of the condition. The study will focus on comparing these pathways between participants carrying the ApoE3 allele, which is more common, and those with the ApoE4 allele, associated with early onset AD. A total of forty participants will be recruited and randomly assigned into two groups with twenty participants each. Blood samples will be collected at the beginning to establish a baseline. One group will receive a daily dose of 30 mL of EVOO for 6 months, while the other will serve as a control. At the end of this period, blood samples will be collected again to assess possible differences that arise from the treatment (Table 4) (Clinicaltrials.gov: NCT05929924). In a distinct study, for which no updates have been found, despite thorough searches, the authors wanted to assess the effects of EVOO “Coratina” in mild cognitive impairment and Alzheimer’s disease patients (EVOCAD). A total of 24 individuals would be recruited, and half would receive EVOO “corantia”, while the other half was administered biophenol low dose olive oil for a year. During this time, clinical assessment would be performed using several examinations to obtain results that could be compared between the groups (Clinicaltrials.gov: NCT04229186). Within the context of a trial entitled Auburn University Research on Olive Oil for Alzheimer’s Disease (Table 4) (AU-ROOAD), the effects of the daily consumption of refined olive oil and EVOO for 6 months were explored in a cohort of 25 participants with MCI. The focus was to address the differences on the permeability of the BBB and brain function, as well as in cognitive function and AD blood biomarkers, which were the secondary outcomes. The brain–blood barrier is commonly compromised in individuals with MCI, leading to increased neurotoxins in the brain, which contributes to disease progression in severe cases of Alzheimer’s and dementia patients with MCI. The results demonstrated that EVOO resulted in decreased BBB permeability and increased brain connectivity, improving overall dementia ratings and behavior. Nevertheless, both EVOO and refined olive oil improved cognitive and behavioral parameters and lowered AD markers, indicating their potential effect on A*β* clearance. This study supports further trials that investigate the potential of olive oil and its bioactive compounds in preventing the progression from MCI to AD (Table 4) [265]. Furthermore, in the MICOIL pilot study, the authors investigated the consequences of administering Greek high-phenolic early harvest EVOO (HP-EH-EVOO), moderate-phenolic EVOO (MP-EVOO), or the Mediterranean diet to individuals with MCI. They were able to conclude that treatment with either HP-EH-EVOO or MP-EVOO resulted in increased cognitive function, with more noticeable effects when patients were treated with HP-EH-EVOO, independently of the ApoE4 genotype [277].

Clinical trials evaluating the effects of olive oil’s by-products on AD appear to be less frequent, as this is an unexplored area, although it possesses exquisite potential. In an ongoing clinical trial (Clinicaltrials.gov: NCT06245616), the authors have built their hypothesis considering previous research that demonstrated that a substantial portion of A*β* peptide is transported by triglyceride-rich lipoproteins (TRL), which are produced in the liver and intestine following a meal [278,279]. After the consumption of fatty meals, TRL levels can temporarily rise in the bloodstream, resulting in postprandial dyslipidemia, a transient but pronounced increase in blood lipids [280]. Furthermore, the literature suggests that the consumption of OP oil during the postprandial period is linked with the production of TLRs that can retain bioactive compounds, which, in turn, can reduce neuroinflammation, a key factor in the progression of AD, by inactivating microglia [278,281]. For this study, 40 early-stage AD patients and 40 healthy individuals were selected and both groups were divided into 2 distinct groups based on their triglyceride levels. All participants were administered both OP oil and high-oleic sunflower oil. While the trial is not yet completed, the hypothesis appears to have promising potential to uncover significant results (Clinicaltrials.gov: NCT06245616). Another ongoing study (GOLDEN) aims to evaluate the effects of olive-leaf beverage in individuals with mild dementia, when compared with the Mediterranean diet (Table 4) (Clinicaltrials.gov: NCT04440020). To our knowledge, these are the only two trials that have considered using olive oil’s by-products in the treatment or prevention of AD.

## 5. Conclusions

The olive oil industry generates substantial amounts of waste, which, as anticipated, leads to a myriad of complications. The by-products of the olive oil industry include olive leaves, OMWW, and OP, and their generation translates into key environmental and economic challenges that need to be addressed. For instance, OP formation involves water evaporation, necessitating prolonged storage under specific conditions, which disrupts operation at olive mills and slows production. In the case of OMWW, the impediments are slightly different, although also noticeable, as it is a toxic byproduct that requires pre-treatment. This not only prolongs the process, but also attracts insects and poses a risk of additional water contamination, if improperly managed. Olive leaves, while rich in phenolic compounds that help in protecting the plant from predators, present a challenge due to the instability of phenolic compounds, which are easily oxidized to originate toxic molecules, such as quinones and polymeric phenols. Hence, it is essential to develop and implement alternatives for the effective management and sustainable exploitation of these by-products, not only minimizing waste, but also promoting the recovery of valuable resources.

Over the last few years, research has shown that the by-products of the olive oil industry possess several compounds with remarkable applications in various contexts. Nevertheless, there are numerous difficulties that need to be overcome, as different origins of extracts and distinct extraction and conservation methods influence the characteristics of the extracts obtained from the by-products. Therefore, the parameters should be adjusted depending on the aim of the study. A common approach has been trying to supplement or create and functional foods that may impact health and contribute to disease prevention.

Polyphenols present neuroprotective effects due to the presence of aromatic rings and hydroxyl groups, which allow them to exert antioxidant properties, specifically scavenging ROS, chelating metal ions, and modulating signaling pathways that can attenuate both oxidative stress and inflammation. This is particularly relevant in the context of AD, a neurodegenerative disorder that affects millions of individuals worldwide and is associated with cognitive decline and memory loss and, ultimately, loss of independence. AD is essentially characterized by extracellular deposition of A*β* plaques and intracellular accumulation of NFTs composed of hyperphosphorylated tau protein.

In in vitro and vivo studies, polyphenols derived from olive oil or olive oil by-products, particularly OLE and HT, showed remarkable effects upon AD models. Nevertheless, it seems fundamental to mention that as a complex disease, AD involves several pathways and factors which complicates the task of attributing observed benefits solely to polyphenol intake. Moreover, the lack of standardized methods and consistent protocols across studies makes it difficult to compare results. These challenges underscore the need for additional research to fully assess the potential of these compounds in preventing or treating AD, particularly human clinical trials.

Clinical trials on AD involving polyphenols, particularly those derived from olive oil by-products, remain relatively scarce. This presents a notable gap in research, given the promising neuroprotective effects associated with these compounds. Future research could benefit significantly from exploring these polyphenols, through the conduction of well-designed trials. These studies could not only advance our understanding of their health impact, but also support sustainability. This approach could contribute to both environmental sustainability and the development of new therapeutic strategies for AD.

## Figures and Tables

**Table 4:** Summary of clinical studies evaluating the effects of olive oil and derivatives on cognitive functions.

Study Title	Participants	Intervention	Objective	Clinical Trial ID
Does extra virgin olive oil induce gene and metabolic changes in healthy subjects	40 (healthy with family history of Alzheimer’s disease	30 mL extra virgin olive oil daily for 6 months	Assess the impact of extra virgin olive oil on the molecular and biological mechanisms associated with AD in healthy individuals with a family history of the disease.	NCT05929924
Pilot Study About Extra Virgin Olive Oil “Coratina” in Mild Cognitive Impairment and Alzheimer’s Disease Patients (EVOCAD)	24 mild cognitive impairment and Alzheimer’s disease patients	Extra virgin olive oil ‘Coratina’ vs. biophenol low-dose olive oil for 12 months	Assess and compare the effects of extra virgin olive oil and biophenol low-dose olive oil on brain and cardiovascular functions.	NCT04229186
Auburn University research on olive oil for Alzheimer’s disease (AU-ROOAD)	24 Mild cognitive impairment patients	Extra-virgin olive oil that is rich with oleocanthal versus phenols and olive oil low in oleocanthal and other phenols, daily for 6 months	Evaluate the effect of extra-virgin olive oil in MCI participants and compare its effect with refined olive oil (ROO; null in phenolic fraction) using a variety of metrics: (i) the blood brain barrier integrity obtained from contrast-enhanced MRI imaging, and brain function and network connectivity obtained by functional MRI (fMRI), (ii) cognitive function assessed using neuropsychological evaluation, and (iii) blood A*β*, tau, phospho-tau 181, and neurofilament light levels.	NCT03824197
Greek high-phenolic early harvest extra virgin olive oil in mild cognitive impairment: the MICOIL Pilot Study	50 mild cognitive impairment patients	High phenolic early-harvest extra virgin olive oil (HP-EH-EVOO) versus moderate-phenolic (MP-EVOO), 50 mL/day, for 12 months	Evaluate the beneficial effect of extra virgin olive oil in comparison to freshly pressed extra virgin olive oil on patients diagnosed with mild cognitive impairment	NCT03362996
Attenuation of Postprandial Inflammatory Processes in Alzheimer’s Disease Patients by Consumption of Pomace Oil (CORDIAL)	80 (40 early-stage Alzheimer’s disease patients, 40 healthy individuals	Olive pomace oil versus high-oleic sunflower oil in the other	Evaluate the effect on triglyceride-rich lipoproteins and neuroinflammation via microglia inactivation	NCT06245616
Management of dementia with olive oil leaves (GOLDEN)	100 mild cognitive impairment patients	Beverage of olive oil from leaves versus dietary supplement: Mediterranean diet, for 24 months	Evaluate and compare the effects of the olive-leaf beverage and Mediterranean diet on memory and cognitive function in individuals with mild dementia.	NCT04440020

## Data Availability

Not applicable.
